# Ramp Sequence May Explain Synonymous Variant Association with Alzheimer’s Disease in the Paired Immunoglobulin-like Type 2 Receptor Alpha (PILRA)

**DOI:** 10.3390/biomedicines13030739

**Published:** 2025-03-18

**Authors:** Justin B. Miller, J. Anthony Brandon, Lauren M. Harmon, Hady W. Sabra, Chloe C. Lucido, Josue D. Gonzalez Murcia, Kayla A. Nations, Samuel H. Payne, Mark T. W. Ebbert, John S. K. Kauwe, Perry G. Ridge

**Affiliations:** 1 Department of Pathology and Laboratory Medicine, University of Kentucky, Lexington, KY 40506, USA; 2 Sanders-Brown Center on Aging, University of Kentucky, Lexington, KY 40506, USAmark.ebbert@uky.edu (M.T.W.E.); 3 Department of Microbiology, Immunology, and Molecular Genetics, University of Kentucky, Lexington, KY 40506, USA; 4 Division of Biomedical Informatics, Department of Internal Medicine, University of Kentucky, Lexington, KY 40506, USA; 5 Department of Biology, Brigham Young University, Provo, UT 84602, USA; lauren.harmon@vai.edu (L.M.H.); josuegon.mur@gmail.com (J.D.G.M.); kauwe@byu.edu (J.S.K.K.); 6 Department of Neuroscience, University of Kentucky, Lexington, KY 40506, USA

**Keywords:** Alzheimer’s disease, ramp sequence, genetic association, codon usage bias, disease association

## Abstract

**Background:** The synonymous variant *NC_000007.14:g.100373690T>C* (*rs2405442:T>C*) in the Paired Immunoglobulin-like Type 2 Receptor Alpha (*PILRA*) gene was previously associated with decreased risk for Alzheimer’s disease (AD) in genome-wide association studies, but its biological impact is largely unknown. **Objective:** We hypothesized that *rs2405442:T>C* decreases mRNA and protein levels by destroying a ramp of slowly translated codons at the 5′ end of *PILRA*. **Methods:** We assessed *rs2405442:T>C* predicted effects on *PILRA* through quantitative polymerase chain reactions (qPCRs) and enzyme-linked immunosorbent assays (ELISAs) using Chinese hamster ovary (CHO) cells. RESULTS: Both mRNA (*p* = 1.9184 × 10^−13^) and protein (*p* = 0.01296) levels significantly decreased in the mutant versus the wildtype in the direction that we predicted based on the destruction of a ramp sequence. **Conclusions:** We show that *rs2405442:T>C* alone directly impacts *PILRA* mRNA and protein expression, and ramp sequences may play a role in regulating AD-associated genes without modifying the protein product.

## 1. Introduction

Alzheimer’s disease (AD) is highly heritable, with genetic variants accounting for 58–79% of total dementia risk [1]. Common genetic effects identified through genome-wide association studies (GWASs) implicate approximately 80 genetic risk loci with AD-type dementia [2,3,4,5,6,7,8,9], yet less is known about which genetic variants drive disease association. Many factors from high-impact diseases in addition to AD (i.e., amyloid plaques and neurofibrillary tangles) contribute to the dementia phenotype [10,11], and heterogeneity plays a role in several distinct subtypes based on biomarkers [12,13,14], genetics [15,16], imaging [13,17,18,19,20,21], and impact on daily function [22,23]. Similarly, clinical symptoms of dementia are heterogeneous and based on a progression of amyloid deposition, tau buildup, and neurodegeneration (A/T/N) [24], with mixed pathologies impacting the speed of cognitive decline [13,17,22,25,26,27,28,29]. While polygenic risk scores (PRSs) have recently emerged as a viable tool to aggregate genetic risk across various disease-associated loci so that complex population-specific genetic interactions can be simplified to a single risk score [30,31], they do not attempt to characterize the biological mechanisms underpinning disease associations, and many associations have yet to be biologically validated. One of those currently unsubstantiated associations is located in the Paired Immunoglobulin-like Type 2 Receptor Alpha (*PILRA*). Here, we biologically assessed the effects of the synonymous variant, *NC_000007.14:g.100373690T>C* (*rs2405442:T>C)*, and propose that its association with AD is caused by the destruction of a ramp of slowly translated codons at the 5′ end of *PILRA*.

Ramp sequences are essential genetic regulatory regions that counterintuitively maximize overall translational efficiency by slowing translation at the 5′ end of genes to evenly space ribosomes, which limits downstream ribosomal collisions and reduces translational errors [32,33,34,35,36,37,38,39]. Specifically, ramp sequences increase mRNA stability and gene expression, especially in genes that have higher ribosome density, higher mRNA levels, and a strong correlation between mRNA and protein expression [38,40] by reducing ribosome stalling and mRNA degradation via ribosome-associated protein quality control (RQC) [41]. Ramp sequences are phylogenetically conserved [42], yet differ between human populations [43] and cell types [44], which corresponds with population and cell-specific differences in gene expression [38,43,44]. A ramp sequence is present in *PILRA*, which likely helps regulate both protein and mRNA levels within different cell types.

*PILRA* is an inhibitory receptor that regulates immune cells [45] of the myelomonocytic lineage such as macrophages, dendritic cells, monocytes and monocyte-derived dendritic cells and is highly expressed in the lymph node and neural tissues [46,47]. It functions by negatively regulating neutrophil infiltration and controlling monocyte mobility [45,48]. The innate and adaptive immune responses have been implicated in AD [49], and gene regulation of *PILRA*-expressing myeloid cells have also been associated with AD [50]. AD risk alleles are specifically enriched in active enhancers of myeloid-derived cells that express *PILRA* such as monocytes, macrophages, and microglia, with *PILRA* expression contributing to a systemic failure of cell-mediated amyloid-β (Aβ) clearance [51], which likely contributes to AD onset and progression.

Several studies have found AD-associated variants in *PILRA* to be protective [7,52,53], yet the protective variant effects are generally attributed to a missense variant, *NC_000007.14:g.100374211A>G* (*rs1859788:A>G*) [53], which is in high linkage disequilibrium with *rs2405442:T>C*. However, we show that *rs2405442:T>C* alone disrupts the *PILRA* ramp sequence by increasing codon adaptiveness relative to the rest of the transcript, which in turn significantly decreases both mRNA (*p* = 1.9184 × 10^−13^) and protein (*p* = 0.01296) levels in the direction that we hypothesized based on predicted ramp sequence effects. This study is the first time where ramp sequences have been used to prioritize disease-associated variants for biological validation and offers a likely biological mechanism that can regulate *PILRA* expression without altering the final protein product. Further, these analyses show that the synonymous variant *rs2405442:T>C* alone disrupts *PILRA* and may drive association with AD.

## 2. Materials and Methods

### 2.1. Identifying AD-Associated Genetic Variants

We prioritized genetic variants for ramp sequence analyses using the GWAS summary statistics from Jansen, Savage [54] because they report all single nucleotide polymorphism (SNP) associations with AD that exceeded the genome-wide significance threshold of *p* ≤ 5.0 × 10^−8^ before accounting for linkage disequilibrium at each locus. While Bellenguez, Küçükali [2] report additional genetic associations with AD, we opted to not include their summary statistics in these analyses because they report only variants that are likely independent hits after performing linkage disequilibrium analyses, which greatly reduces the number of reported genetic associations by using *p*-values to prioritize the independent hits. In some cases, leading variants are chosen based on predicted effects, which would also bias our analyses since variant-level ramp sequence effects have not previously been reported. Additionally, ramp sequences are affected by only exonic coding variants, which are generally rarer than intronic variants in GWASs and may be missed by a clumping approach to choose independent hits. Thus, we decided that the full table of variant-level associations reported by Jansen, Savage [54] was most appropriate to assess how ramp sequences potentially impact AD. Although 2357 variants were originally reported [54], only 51 variants were reported in exonic regions, with only 14 SNPs identified as plausible causal variants based on a fine-mapping model that accounts for *APOE* ε4, roughly corresponding to *p* > ~2.0 × 10^−4^. All computational analyses were limited to those 14 variants.

Ensembl [55] web queries were conducted in December 2023 to obtain the most severe variant consequence, highest minor allele frequency, Combined Annotation Dependent Depletion (CADD) score [56], Genomic Evolutionary Rate Profiling (GERP) score [57], and GRCh38.p14 reference genome chromosome coordinates. RegulomeDB [58] scores were then queried using the RegulomeDB web interface. All transcript isoforms with GRCh38.p14 reference genome coordinates were downloaded from the National Center for Biotechnology Information (NCBI; https://www.ncbi.nlm.nih.gov/datasets/genome/GCF_000001405.40/) accessed on 1 December 2023.

### 2.2. Identifying Ramp Sequences

Since ramp sequences are dependent on tissue and cell-specific tRNA pools, we used The Ramp Atlas [44] to download pre-computed tRNA efficiency values for 62 human tissues included in a consensus dataset derived from the Genotype-Tissue Expression (GTEx) Project [59], Functional ANnoTation of the Mammalian genome (FANTOM5) [60], and the Human Protein Atlas [47] databases. An additional file consisting of codon efficiencies from 66 cell types was also downloaded from The Ramp Atlas and used to analyze cell-specific effects on *PILRA* ramp sequences. The relative codon adaptiveness in each tissue or cell type could impact the presence or absence of a ramp sequence by changing where translational bottlenecks occur without altering the DNA sequence. We used ExtRamp [36] to identify ramp sequences in the reference and mutant sequence for each tissue or cell type individually using the -a option to specify the relative codon adaptiveness for each tissue or cell, which resulted in 256 total ramp sequence calculations for each variant (62 tissues + 66 cell types for both the reference and mutant sequences). By default, ExtRamp identifies ramp sequences based on codon translational efficiencies spanning nine codons, which is roughly the size of a ribosome window [61]. The harmonic mean is then used to determine the translational rate within that window, which is compared to the harmonic mean translational efficiency of the entire gene sequence. True outlier regions that occur at the 5′ end of genes are considered ramp sequences and were reported for each tissue and cell type. All scripts used to identify ramp sequences are available at https://github.com/jmillerlab/PILRA_ramp.

### 2.3. Biological Assessment of Ramp Sequence Effects in PILRA

The synonymous variant *rs2405442:T>C* in *PILRA* was the only AD-associated variant predicted to destroy a ramp sequence. Since all five *PILRA* isoforms are predicted to have ramp sequences, we opted to use the longest *PILRA* isoform (Ensembl accession: *ENST00000198536.7*; NCBI accession: *NM_013439.3*) to assess *rs2405442:T>C* effects on mRNA and protein levels. DNA sequences for *ENST00000198536.7* (wildtype) and *ENST00000198536.7* containing *rs2405442:T>C* (mutant) were synthesized by GenScript Biotech. A Human c-Myc proto-oncogene (*MYC*) epitope tag, FLAG^®^ epitope tag, and enterokinase cleavage site were attached to the 3′ end of the coding sequences. The reference and mutant sequences with annotated features are depicted in Supplementary Figures S1 and S2.

Three independent replicates of quantitative polymerase chain reactions (qPCRs), each of which contained eight technical replicates, were used to assess how the synonymous variant, *rs2405442:T>C*, impacted *PILRA* mRNA levels in both the mutant and the wildtype transfected cells. Similarly, three independent sets of eight technical replicates were used to assess how PILRA protein levels differed between the mutant and wildtype using Enzyme-Linked Immunosorbent Assay (ELISA). Detailed instructions for replicating each protocol are described below.

### 2.4. Transfection of Wildtype and Mutant Transcripts

The wildtype and mutant sequences synthesized by GenScript Biotech were each inserted into separate mammalian expression vectors pCMV6-AN-myc-DDK (ORIGENE, Catalog #PS100016). Transformation protocols were followed as recommended by the manufacturer. In brief, plasmids were transformed into competent DH5a cells, amplified, and purified using the ZymoPURE II Plasmid Maxiprep kit (Catalog #D4203). Purified wildtype and mutant plasmids were then transfected into Chinese Hamster Ovary-K1 (CHO-K1) cells using the Lipofectamine™ 3000 Transfection Reagent protocol (ThermoFisher, Waltham, MA, USA, Catalog #15338100). Properly transfected cells were selected using the antibiotic G418 sulfate (ThermoFisher, Catalog #10131035). Transfected cells were grown in F12 media (ThermoFisher, Catalog #11765054) with 10 mg/mL penicillin, 10 μg/mL streptomycin (Gibco, Catalog #21127-022), and 10% FBS (HYCLone Catalog #SH30071.01). Cell media was changed every 48 to 72 h depending on cell confluency levels. CHO-K1 transfected cells were then used in both the qPCR and ELISA protocols.

### 2.5. qPCR Protocol

Total RNA was extracted from the mutant and the wildtype CHO-K1 transfected cells using the SPLIT RNA Extraction Kit (Lexogen, Vienna, Wien, Austria, Catalog #008) and following the manufacturer guidelines. When the total RNA was purified and ready for quality control, the total RNA concentration was quantified using a NanoDrop spectrophotometer and the Agilent DNF-471 RNA Kit 15nt (Agilent, Santa Clara, CA, USA, Catalog #DNF-471-0500). Reverse transcription of the RNA into complementary DNA (cDNA) was then performed to convert the RNA molecules into their corresponding cDNA sequences using the High-Capacity cDNA Reverse Transcription Kit (Thermo Fisher Scientific, Waltham, MA, USA, Catalog #4374966). We then performed qPCR (PerfeCTa SYBR^®^ Green SuperMix, Quantabio Beverly, MA, USA, Catalog #95054-500) to quantify gene expression levels, and cDNA concentration was quantified using the Agilent Femto Pulse. Forward and reverse primers (respectively GTAAAACGACGGCCAGT and ACTGGCCGTCGTTTTAC) were ordered from Life Technologies Corporation in March 2023. *PILRA* mRNA expression was normalized to total RNA to account for potential differences in qPCR amplification efficiency between tests. We calculated the relative expression of *PILRA* (∆C_t_) by subtracting the *PILRA* counts (C_t_) from a housekeeping gene, Glyceraldehyde 3-phosphate dehydrogenase (*GAPDH*; i.e., *PILRA−GAPDH*). True outliers were then removed to limit potential technical artifacts. Finally, we calculated the fold change in expression (∆∆C_t_) [62] using the following equation with the average ∆C_t MUTANT_ and ∆C_t CONTROL_ across all replicates: ∆∆C_t_ = 2^−(∆Ct MUTANT−∆Ct CONTROL)^.

### 2.6. ELISA Protocol

Proteins were extracted from the mutant and the wildtype CHO-K1 transfected cells, and the total protein concentration was quantified using the Pierce™ BCA Protein Assay (Thermo Fisher Scientific, Catalog #23225 and 23227) following the manufacturer guidelines. The human PILRA protein concentration was quantitively measured in the mutant and the wildtype CHO-K1 transfected cells using the Human PILR-alpha ELISA Kit (Thermo Fisher Scientific, Catalog #EH368RB) by following the manufacturer guidelines. The concentration of PILRA was then normalized to the total concentration of proteins to account for potential variation between tests.

## 3. Results

### 3.1. Ramp Sequence Variation Caused by Exonic GWAS Hits

Table 1 lists the 14 credibly causal exonic SNPs spanning 12 genes and 79 isoforms reported by Jansen, Savage [54]. Each SNP was previously associated with AD and is here reported with the following: the CADD [56] score from GRCh38-v1.6; highest MAF reported in Ensembl [55] from any population in 1000 G Phase 3 [63], NHLBI Exome Sequencing Project [64], and gnomAD [65]; the GERP [57] score from 91_mammals.gerp_conservation_score; RegulomeDB [58] score; and effect on ramp sequences. Six SNPs (*rs2405442:T>C*, *rs12453:T>C*, *rs7982:A>G*, *rs1859788:A>G*, *rs12459419:C>T*, and *rs2296160:A>G*) are in five genes (*PILRA*, *MS4A6A*, *CR1*, *CLU*, and *CD33*) with ramp sequences. While *rs2405442:T>C*, *rs12453:T>C*, *rs1859788:A>G*, and *rs12459419:C>T* change the ramp sequence length, only *rs2405442:T>C* has a severe impact on ramp sequences by destroying it in at least one tissue or cell type.

### 3.2. PILRA Ramp Sequence

**Figure 1:**
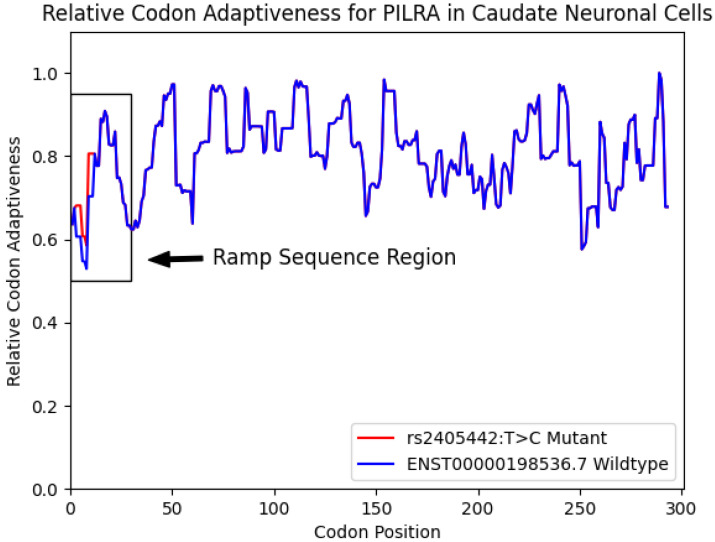
Relative codon adaptiveness for *PILRA* in caudate neuronal cells. Figure 1 shows the relative codon adaptiveness of the longest PILRA reference isoform and the mutant gene averaged over a nine-codon window in caudate neuronal cells. The mutant gene (*rs2405442:T>C*) has a higher codon adaptiveness at the beginning of the gene sequence compared to the wildtype gene.

Using ExtRamp, we calculated the relative codon adaptiveness of *PILRA* using all four isoforms in GRCh38. We then calculated the relative codon adaptiveness of *PILRA* with the synonymous variant *rs2405442:T>C* and found that a ramp sequence is present in the reference isoforms but not the mutant isoforms for all four transcripts. The *PILRA* SNP, *rs2405442:T>C*, increases regional mean translational efficiency at the 5′ end of *PILRA*, effectively destroying the ramp sequence (see Figure 1).

Using the consensus dataset consisting of gene expression from GTEx, FANTOM5, and the Human Protein Atlas, *PILRA* has a ramp sequence in 26/62 tissues. Using a single-cell dataset from the Human Protein Atlas, we also predicted that ramp sequences occur in *PILRA* in 20/66 cell types. The synonymous variant *rs2405442:T>C* destroyed the ramp sequence in all 46 tissues and cell types that normally contain a ramp sequence. Table 2 lists the 46 tissues or cell types with ramp sequences in *PILRA* that are affected by *rs2405442:T>C* (see Supplementary Table S1 for tissues and cell types without a *PILRA* ramp sequence). Specific neural cells that lost their ramp sequences include cerebellum Purkinje, hippocampus glial, caudate glial, and caudate neuronal cells. Lymphatic tissues and cells that lost their ramp sequences include the dendritic cells, monocytes, appendix lymphoid tissue, lymph node non-germinal center cells, and spleen cells in the red and white pulps.

In addition to ramp sequences, we also evaluated the effects of *rs2405442:T>C* on other codon usage biases such as the GC content, codon pairing [66], codon aversion [43,67,68,69], and codon translational speed. *PILRA* was not previously identified as having splicing quantitative trait loci (sQTLs) [70], so *rs2405442:T>C* is not predicted to impact splicing. The GC content is slightly increased in the mutant, which would normally indicate higher mRNA expression [71]. Additionally, *rs2405442:T>C* affects the twelfth codon in *PILRA*, changing it from an uncommon leucine-encoding codon, TTG, to the most common leucine-encoding codon, CTG, which would normally indicate a higher translational speed since common codons are generally translated faster than rare codons [72]. Similarly, identical codon pairing suggests that *rs2405442:T>C* would increase translational speed [66] since the mutation increases CTG codon pairing in the transcript from six instances in the wildtype to seven instances in the mutant. Since the synonymous variant does not change the amino acid sequence, co-tRNA codon pairings (i.e., co-occurring amino acid residues) [73] were not assessed. Based on codon usage biases, the ramp sequence indicates decreased mRNA and protein expression while the GC content and codon pairing would suggest increased mRNA and protein expression in the mutant versus the wildtype.

Since the synonymous variant *rs2405442:T>C* is the only credible causal synonymous variant with a predicted deleterious effect on a ramp sequence and is highly associated with AD, it was a good candidate for biological validation. We predicted that the destroyed ramp sequence would have an outsized effect due to ribosome-associated protein quality control induced by increased ribosome collisions [41], and we experimentally validated the predicted effects of *rs2405442:T>C* on *PILRA* mRNA and protein levels with qPCR and ELISA using CHO cells harboring the synonymous variant compared to wildtype cells without the variant.

**Figure 2:**
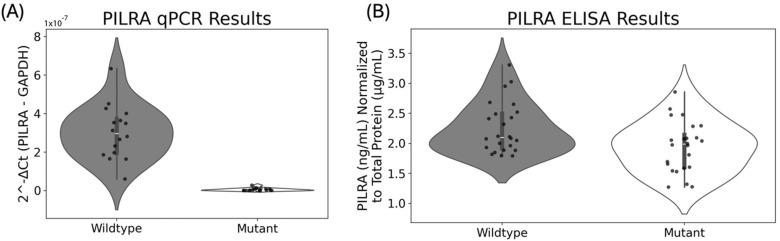
*rs2405442:T>C*’s effects on mRNA and protein levels in CHO cells harboring the synonymous variant compared to wildtype cells without the variant. Three independent sets of eight technical replicates were used to assess differences between the wildtype and the mutant using both qPCR and ELISA. (**A**) shows that *PILRA* mRNA levels are significantly lower in the mutant than the wildtype (*p* = 1.9184 × 10^−13^). Since high C_t_ values show lower expression, we converted the raw C_t_ values to the relative expression by using the formula 2^−Ct^, where C_t_ is the normalized expression of C*_PILRA_*−C*_GAPDH_*. While it is unclear why the wildtype exhibited larger variance than the mutant, all qPCR measurements from the mutant were lower than all measurements from the wildtype, indicating that the mutant decreased mRNA levels compared to the wildtype. Two outliers with higher expression from the wildtype were removed, but did not affect the conclusions; (**B**) shows that *PILRA* protein levels are also significantly lower in the mutant than the wildtype (*p* = 0.01296).

Figure 2 shows that mRNA levels are significantly lower in the mutant than the wildtype (*p* = 1.9184 × 10^−13^). The fold change in expression (∆∆C_t_) is ~131× higher in the wildtype than the mutant. Similarly, protein levels are also significantly higher in the wildtype than the mutant (*p* = 0.01296), with *PILRA* protein levels being, on average, 1.1635× higher in the wildtype cells than the mutant cells. Although the synonymous variant *rs2405442:T>C* has no effect on *PILRA* amino acid residues, it significantly decreases both mRNA and protein levels in the mutant versus the wildtype.

## 4. Discussion

Here, we provide a mechanistic explanation for the association of *rs2405442:T>C* with AD, including experimental validation of its biological effects. This study is the first time where ramp sequences have been used to prioritize disease-associated variants for biological validation.

We recognize that additional biological validation is needed to fully assess the impact of *rs2405442:T>C* on mRNA and protein levels. Additional validation using the Western blot, ribosome profiling, disome-seq, and different cell lines could provide further evidence to support the observed impact of this synonymous variant on *PILRA*. However, this study is the first to quantifiably assess the impact of *rs2405442:T>C* and provides strong evidence that *rs2405442:T>C* alone can impact *PILRA* mRNA and protein levels. Those direct effects may explain its previous association with Alzheimer’s disease, suggesting that *rs2405442:T>C* should not be discounted simply because it is a synonymous variant.

Since reduced *PILRA* inhibitory signaling has previously been shown to induce a protective effect against AD via reduced inhibitory signaling in microglia [53], it is likely that less *PILRA* expression induced by *rs2405442:T>C* would similarly reduce inhibition of immune cells and result in more efficient cell-mediated clearance of Aβ. Although *rs2405442:T>C* creates a more common codon that increases the GC content and codon pairing, which would generally increase mRNA and protein expression, the destroyed ramp sequence seems to outweigh the other codon usage biases producing the observed effects. The destruction of the ramp sequence is expected to increase the frequency of ribosomal collisions, leading to stalled proteins and triggering the recruitment of the ribosome-associated protein quality control [41,74] to degrade aberrant *PILRA* peptides.

*PILRA* gene expression in vivo is also likely affected by the distribution of tissue-specific mature tRNA pools. Some evidence suggests that mature tRNA pools change with environmental factors such as aging, stress, and diet, which would also change the relative codon adaptiveness and presence of a ramp sequence [75]. We show that *rs2405442:T>C* alone can significantly affect mRNA and protein levels independent of other genetic variants or changes in the tRNA pool and further provide a workflow to perform tissue and cell-specific computational analyses to investigate how a genetic variant impact gene-specific ramp sequences in different tissues and cells based on tRNA pool availability. Our tissue and cell-specific data show that after acquiring *rs2405442:T>C*, neural tissues such as caudate glial and neuronal cells, cerebellum Purkinje cells, and hippocampus glial cells are predicted to similarly lose their ramp sequences. Many lymphatic and immune-related tissues and cells are likewise expected to lose their *PILRA* ramp sequences after acquiring the synonymous variant, including dendritic cells, monocytes, PBMCs, appendix lymphoid tissue, lymph node non-germinal center cells, spleen cells in the red and white pulps, and tonsil non-germinal center cells. While we did not directly assess *rs2405442:T>C* effects in AD cell lines, we showed that the synonymous variant *rs2405442:T>C* decreases *PILRA* mRNA and protein levels in CHO-K1 cells by destroying a ramp sequence. Since *PILRA* ramp sequence loss similarly occurs in tissues and cell types known to impact AD, we hypothesize that the previously described association between *rs2405442:T>C* and AD may stem from *rs2405442:T>C* reducing *PILRA* inhibitory signaling in those tissues and cells by destroying the ramp sequence.

The synonymous variant *rs2405442:T>C* has a high minor allele frequency (MAF > 0.35 [63,65]), indicating that natural decreases in *PILRA* expression induced by this variant are well-tolerated in the general population. Therefore, ramp-mediated therapeutics targeting *rs2405442:T>C* may be viable methods to mitigate risk for AD and increase cell-mediated Aβ clearance without inducing other off-target effects.

Here, we show that a ramp sequence plays a crucial role in *PILRA* gene regulation, and the synonymous variant *rs2405442:T>C* alone causes a significant decrease in *PILRA* mRNA and protein levels by disrupting that regulatory mechanism. While synonymous variants are often overlooked in genome-wide association studies, they can significantly alter regulatory biases such as ramp sequences that can directly impact gene expression and protein levels. We outline how to analyze variant effects on ramp sequences, and we present a code repository at https://github.com/jmillerlab/PILRA_ramp to facilitate these types of analyses.

## Figures and Tables

**Table 1:** Credible Causal Exonic Variant Effects. Credible causality is defined and reported by Jansen, Savage [54]. “Loss of Ramp” indicates that the ramp sequence was destroyed in at least one transcript, while “Ramp Size” indicates that the length of the ramp sequence changed in at least one transcript. “Gene with Ramp” indicates that the SNP was located outside the ramp region, yet the gene has a ramp sequence in at least one transcript in at least one cell or tissue.

SNP	Chromosome/Position	Nearest Gene	Transcripts with Ramp Sequence	Most Severe Variant Effect	Highest MAF	CADD Score	GERP Score	RegulomeDB Score	Most Severe Ramp Effect
*rs2405442:T>C*	7:100373690	*PILRA*	4/4 (100%)	Synonymous	0.50 (T)	4.238	−2.24	1f	Loss of Ramp
*rs12453:T>C*	11:60178272	*MS4A6A*	8/14 (57%)	Synonymous	0.50 (C)	0.578	−4.07	1f	Ramp Size
*rs1859788:A>G*	7:100374211	*PILRA*	4/4 (100%)	Missense	0.50 (A)	12.85	1.01	1f	Ramp Size
*rs12459419:C>T*	19:51225221	*CD33*	2/6 (33%)	Missense	0.48 (T)	14.75	0.06	1f	Ramp Size
*rs7982:A>G*	8:27604964	*CLU*	1/2 (50%)	Missense	0.49 (A)	0.920	−3.07	1f	Gene with Ramp
*rs2296160:A>G*	1:207621975	*CR1*	5/5 (100%)	Missense	0.35 (A)	0.001	−3.64	7	Gene with Ramp
*rs3752241:C>G*	19:1053525	*ABCA7*	0/18 (0%)	Synonymous	0.29 (G)	3.833	−4.46	1f	N/A
*rs117618017:C>T*	15:63277703	*APH1B*	0/3 (0%)	Missense	0.31 (T)	16.39	−1.84	1f	N/A
*rs429358:T>C*	19:44908684	*APOE*	0/5 (0%)	Missense	0.38 (C)	16.65	2.01	1f	N/A
*rs9268480:C>T*	6:32396067	*BTNL2*	0/2 (0%)	Synonymous	0.35 (T)	3.813	−1.07	1f	N/A
*rs1135173:G>A*	2:233146227	*INPP5D*	0/2 (0%)	Synonymous	0.49 (A)	4.311	−3.25	1f	N/A
*rs157581:T>C*	19:44892457	*TOMM40*	0/4 (0%)	Missense	0.50 (C)	14.60	−1.14	1f	N/A
*rs11556505:C>T*	19:44892887	*TOMM40*	0/4 (0%)	Missense	0.18 (T)	6.035	−6.99	5	N/A
*rs75932628:C>T*	6:41161514	*TREM2*	0/2 (0%)	Missense	0.02 (T)	26.1	NA	2b	N/A

**Table 2:** Tissues and cell types that normally have a ramp sequence in *PILRA*. The ramp sequence is universally destroyed in the mutant containing *rs2405442:T>C*.

Tissues with *PILRA* Ramp Sequence	Cell Types with *PILRA* Ramp Sequence
Amygdala	Appendix lymphoid tissue
Cerebral cortex	Caudate glial
Colon	Caudate neuronal
Corpus callosum	Cerebellum Purkinje
Ductus deferens	Cervix uterine glandular
Duodenum	Dendritic cells
Esophagus	Hippocampus glial
Fallopian tube	Lung pneumocytes
Gallbladder	Lymph node nongerminal center
Heart muscle	Monocytes
Hippocampal formation	Oral mucosa squamous epithelial
Hypothalamus	Pancreas islets of Langerhans
Olfactory region	Prostate glandular
Pancreas	Seminal vesicle glandular
Retina	Skin1 fibroblasts
Salivary gland	Skin keratinocytes
Seminal vesicle	Skin melanocytes
Skeletal muscle	Soft tissue1 fibroblasts
Skin	Spleen cells in red pulp
Small intestine	Spleen cells in white pulp
Spleen	Thyroid gland glandular
Stomach	Tonsil nongerminal center
Tongue	Total PBMC

## Data Availability

All scripts developed and used for these analyses are publicly available at https://github.com/jmillerlab/PILRA_ramp.

## Supplementary Materials

The following supporting information can be downloaded at: https://www.mdpi.com/article/10.3390/biomedicines13030739/s1, Figure S1: Wildtype Sequence with Annotated Features; Figure S2: Mutant Sequence with Annotated Features; Table S1: Tissues and Cell Types Without a Ramp Sequence in PILRA.

## Abbreviations

The following abbreviations are used in this manuscript:

AD	Alzheimer’s disease
CHO	Chinese hamster ovary
RQC	Ribosome-associated protein quality control

## References

[1] Gatz M., Reynolds C.A., Fratiglioni L., Johansson B., Mortimer J.A., Berg S., Fiske A., Pedersen N.L. (2006). Role of Genes and Environments for Explaining Alzheimer Disease. Arch. Gen. Psychiatry.

[2] Bellenguez C., Kucukali F., Jansen I.E., Kleineidam L., Moreno-Grau S., Amin N., Naj A.C., Campos-Martin R., Grenier-Boley B., Andrade V. (2022). New insights into the genetic etiology of Alzheimer’s disease and related dementias. Nat. Genet..

[3] Balin B.J., Hudson A.P. (2014). Etiology and Pathogenesis of Late-Onset Alzheimer’s Disease. Curr. Allergy Asthma Rep..

[4] Marioni R.E., Harris S.E., Zhang Q., McRae A.F., Hagenaars S.P., Hill W.D., Davies G., Ritchie C.W., Gale C.R., Starr J.M. (2018). GWAS on family history of Alzheimer’s disease. Transl. Psychiatry.

[5] Jun G., Naj A.C., Beecham G.W., Wang L.S., Buros J., Gallins P.J., Buxbaum J.D., Ertekin-Taner N., Fallin M.D., Friedland R. (2010). Meta-analysis Confirms CR1, CLU, and PICALM as Alzheimer Disease Risk Loci and Reveals Interactions with APOE Genotypes. Arch. Neurol..

[6] Hu X., Pickering E., Liu Y.C., Hall S., Fournier H., Katz E., Dechairo B., John S., van Eerdewegh P., Soares H. (2011). Meta-Analysis for Genome-Wide Association Study Identifies Multiple Variants at the BIN1 Locus Associated with Late-Onset Alzheimer’s Disease. PLoS ONE.

[7] Lambert J.C., Ibrahim-Verbaas C.A., Harold D., Naj A.C., Sims R., Bellenguez C., DeStafano A.L., Bis J.C., Beecham G.W., Grenier-Boley B. (2013). Meta-analysis of 74,046 individuals identifies 11 new susceptibility loci for Alzheimer’s disease. Nat. Genet..

[8] Ridge P.G., Hoyt K.B., Boehme K., Mukherjee S., Crane P.K., Haines J.L., Mayeux R., Farrer L.A., Pericak-Vance M.A., Schellenberg G.D. (2016). Assessment of the genetic variance of late-onset Alzheimer’s disease. Neurobiol. Aging.

[9] Andrews S.J., Renton A.E., Fulton-Howard B., Podlesny-Drabiniok A., Marcora E., Goate A.M. (2023). The complex genetic architecture of Alzheimer’s disease: Novel insights and future directions. EBioMedicine.

[10] Escott-Price V., Sims R., Bannister C., Harold D., Vronskaya M., Majounie E., Badarinarayan N., Morgan K., Passmore P., Holmes C. (2015). Common polygenic variation enhances risk prediction for Alzheimer’s disease. Brain.

[11] Bakulski K.M., Vadari H.S., Faul J.D., Heeringa S.G., Kardia S.L.R., Langa K.M., Smith J.A., Manly J.J., Mitchell C.M., Benke K.S. (2021). Cumulative Genetic Risk and APOE ε4 Are Independently Associated With Dementia Status in a Multiethnic, Population-Based Cohort. Neurol. Genet..

[12] Bredesen D.E. (2015). Metabolic profiling distinguishes three subtypes of Alzheimer’s disease. Aging.

[13] Ferreira D., Verhagen C., Hernandez-Cabrera J.A., Cavallin L., Guo C.J., Ekman U., Muehlboeck J.S., Simmons A., Barroso J., Wahlund L.O. (2017). Distinct subtypes of Alzheimer’s disease based on patterns of brain atrophy: Longitudinal trajectories and clinical applications. Sci. Rep..

[14] Eppig J.S., Edmonds E.C., Campbell L., Sanderson-Cimino M., Delano-Wood L., Bondi M.W., for the Alzheimer’s Disease Neuroimaging Initiative (2017). Statistically derived subtypes and associations with cerebrospinal fluid and genetic biomarkers in mild cognitive impairment: A latent profile analysis. J. Int. Neuropsychol. Soc..

[15] Squitti R., Ventriglia M., Gennarelli M., Colabufo N.A., El Idrissi I.G., Bucossi S., Mariani S., Rongioletti M., Zanetti O., Congiu C. (2017). Non-ceruloplasmin copper distincts subtypes in Alzheimer’s disease: A genetic study of ATP7B frequency. Mol. Neurobiol..

[16] Mao Y.-F., Guo Z.-Y., Pu J.-L., Chen Y.-X., Zhang B.-R. (2015). Association of CD33 and MS4A cluster variants with Alzheimer’s disease in East Asian populations. Neurosci. Lett..

[17] Mann U.M., Mohr E., Gearing M., Chase T.N. (1992). Heterogeneity in Alzheimer’s disease: Progression rate segregated by distinct neuropsychological and cerebral metabolic profiles. J. Neurol. Neurosurg. Psychiatry.

[18] Na H.K., Kang D.R., Kim S., Seo S.W., Heilman K.M., Noh Y., Na D.L. (2016). Malignant progression in parietal-dominant atrophy subtype of Alzheimer’s disease occurs independent of onset age. Neurobiol. Aging.

[19] Park J.Y., Na H.K., Kim S., Kim H., Kim H.J., Seo S.W., Na D.L., Han C.E., Seong J.K., Alzheimer’s Disease Neuroimaging Initiative (2017). Robust Identification of Alzheimer’s Disease subtypes based on cortical atrophy patterns. Sci. Rep..

[20] Persson K., Eldholm R.S., Barca M.L., Cavallin L., Ferreira D., Knapskog A.B., Selbaek G., Braekhus A., Saltvedt I., Westman E. (2017). MRI-assessed atrophy subtypes in Alzheimer’s disease and the cognitive reserve hypothesis. PLoS ONE.

[21] Varol E., Sotiras A., Davatzikos C. (2017). HYDRA: Revealing heterogeneity of imaging and genetic patterns through a multiple max-margin discriminative analysis framework. NeuroImage.

[22] Mukherjee S., Mez J., Trittschuh E.H., Saykin A.J., Gibbons L.E., Fardo D.W., Wessels M., Bauman J., Moore M., Choi S.-E. (2018). Genetic data and cognitively defined late-onset Alzheimer’s disease subgroups. Mol. Psychiatry.

[23] Warren J.D., Fletcher P.D., Golden H.L. (2012). The paradox of syndromic diversity in Alzheimer disease. Nat. Rev. Neurol..

[24] Jack C.R., Bennett D.A., Blennow K., Carrillo M.C., Feldman H.H., Frisoni G.B., Hampel H., Jagust W.J., Johnson K.A., Knopman D.S. (2016). A/T/N: An unbiased descriptive classification scheme for Alzheimer disease biomarkers. Neurology.

[25] Bondareff W., Mountjoy C.Q., Roth M., Rossor M.N., Iversen L.L., Reynolds G.P. (1987). Age and histopathologic heterogeneity in Alzheimer’s disease: Evidence for subtypes. Arch. Gen. Psychiatry.

[26] Crane P.K., Trittschuh E., Mukherjee S., Saykin A.J., Sanders R.E., Larson E.B., McCurry S.M., McCormick W., Bowen J.D., Grabowski T. (2017). Incidence of cognitively defined late-onset Alzheimer’s dementia subgroups from a prospective cohort study. Alzheimer’s Dement..

[27] Cummings J.L. (2000). Cognitive and behavioral heterogeneity in Alzheimer’s disease: Seeking the neurobiological basis. Neurobiol. Aging.

[28] Larner A., Doran M. (2006). Clinical phenotypic heterogeneity of Alzheimer’s disease associated with mutations of the presenilin–1 gene. J. Neurol..

[29] Pillon B., Dubois B., Lhermitte F., Agid Y. (1986). Heterogeneity of cognitive impairment in progressive supranuclear palsy, Parkinson’s disease, and Alzheimer’s disease. Neurology.

[30] Purcell S.M., Wray N.R., Stone J.L., Visscher P.M., O’Donovan M.C., Sullivan P.F., Sklar P., Purcell S.M., Stone J.L., Sullivan P.F. (2009). Common polygenic variation contributes to risk of schizophrenia and bipolar disorder. Nature.

[31] Lewis C.M., Vassos E. (2020). Polygenic risk scores: From research tools to clinical instruments. Genome Med..

[32] Dittmar K.A., Goodenbour J.M., Pan T. (2006). Tissue-specific differences in human transfer RNA expression. PLoS Genet..

[33] Waldman Y.Y., Tuller T., Shlomi T., Sharan R., Ruppin E. (2010). Translation efficiency in humans: Tissue specificity, global optimization and differences between developmental stages. Nucleic Acids Res..

[34] Tuller T., Zur H. (2015). Multiple roles of the coding sequence 5’ end in gene expression regulation. Nucleic Acids Res..

[35] Verma M., Choi J., Cottrell K.A., Lavagnino Z., Thomas E.N., Pavlovic-Djuranovic S., Szczesny P., Piston D.W., Zaher H.S., Puglisi J.D. (2019). A short translational ramp determines the efficiency of protein synthesis. Nat. Commun..

[36] Miller J.B., Brase L.R., Ridge P.G. (2019). ExtRamp: A novel algorithm for extracting the ramp sequence based on the tRNA adaptation index or relative codon adaptiveness. Nucleic Acids Res..

[37] Tuller T., Veksler-Lublinsky I., Gazit N., Kupiec M., Ruppin E., Ziv-Ukelson M. (2011). Composite effects of gene determinants on the translation speed and density of ribosomes. Genome Biol..

[38] Tuller T., Carmi A., Vestsigian K., Navon S., Dorfan Y., Zaborske J., Pan T., Dahan O., Furman I., Pilpel Y. (2010). An Evolutionarily Conserved Mechanism for Controlling the Efficiency of Protein Translation. Cell.

[39] Dana A., Tuller T. (2014). The effect of tRNA levels on decoding times of mRNA codons. Nucleic Acids Res..

[40] Park H., Subramaniam A.R. (2019). Inverted translational control of eukaryotic gene expression by ribosome collisions. PLoS Biol..

[41] Joazeiro C.A.P. (2019). Mechanisms and functions of ribosome-associated protein quality control. Nat. Rev. Mol. Cell Biol..

[42] McKinnon L.M., Miller J.B., Whiting M.F., Kauwe J.S.K., Ridge P.G. (2021). A comprehensive analysis of the phylogenetic signal in ramp sequences in 211 vertebrates. Sci. Rep..

[43] Hodgman M.W., Miller J.B., Meurs T.E., Kauwe J.S.K. (2020). CUBAP: An interactive web portal for analyzing codon usage biases across populations. Nucleic Acids Res..

[44] Miller J.B., Meurs T.E., Hodgman M.W., Song B., Miller K.N., Ebbert M.T.W., Kauwe J.S.K., Ridge P.G. (2022). The Ramp Atlas: Facilitating tissue and cell-specific ramp sequence analyses through an intuitive web interface. NAR Genom. Bioinform..

[45] Wang J., Shiratori I., Uehori J., Ikawa M., Arase H. (2013). Neutrophil infiltration during inflammation is regulated by PILRα via modulation of integrin activation. Nat. Immunol..

[46] Uhlén M., Fagerberg L., Hallström B.M., Lindskog C., Oksvold P., Mardinoglu A., Sivertsson Å., Kampf C., Sjöstedt E., Asplund A. (2015). Tissue-based map of the human proteome. Science.

[47] Pontén F., Jirström K., Uhlen M. (2008). The Human Protein Atlas—A tool for pathology. J. Pathol..

[48] Kohyama M., Matsuoka S., Shida K., Sugihara F., Aoshi T., Kishida K., Ishii K.J., Arase H. (2016). Monocyte infiltration into obese and fibrilized tissues is regulated by PILRα. Eur. J. Immunol..

[49] Selkoe D.J., Hardy J. (2016). The amyloid hypothesis of Alzheimer’s disease at 25 years. EMBO Mol. Med..

[50] Huang K.-l., Marcora E., Pimenova A.A., Di Narzo A.F., Kapoor M., Jin S.C., Harari O., Bertelsen S., Fairfax B.P., Czajkowski J. (2017). A common haplotype lowers PU.1 expression in myeloid cells and delays onset of Alzheimer’s disease. Nat. Neurosci..

[51] Li Y., Laws S.M., Miles L.A., Wiley J.S., Huang X., Masters C.L., Gu B.J. (2021). Genomics of Alzheimer’s disease implicates the innate and adaptive immune systems. Cell Mol. Life Sci..

[52] Patel T., Brookes K.J., Turton J., Chaudhury S., Guetta-Baranes T., Guerreiro R., Bras J., Hernandez D., Singleton A., Francis P.T. (2018). Whole-exome sequencing of the BDR cohort: Evidence to support the role of the PILRA gene in Alzheimer’s disease. Neuropathol. Appl. Neurobiol..

[53] Rathore N., Ramani S.R., Pantua H., Payandeh J., Bhangale T., Wuster A., Kapoor M., Sun Y., Kapadia S.B., Gonzalez L. (2018). Paired Immunoglobulin-like Type 2 Receptor Alpha G78R variant alters ligand binding and confers protection to Alzheimer’s disease. PLoS Genet..

[54] Jansen I.E., Savage J.E., Watanabe K., Bryois J., Williams D.M., Steinberg S., Sealock J., Karlsson I.K., Hägg S., Athanasiu L. (2019). Genome-wide meta-analysis identifies new loci and functional pathways influencing Alzheimer’s disease risk. Nat. Genet..

[55] Harrison P.W., Amode M.R., Austine-Orimoloye O., Azov A.G., Barba M., Barnes I., Becker A., Bennett R., Berry A., Bhai J. (2024). Ensembl 2024. Nucleic Acids Res..

[56] Rentzsch P., Witten D., Cooper G.M., Shendure J., Kircher M. (2019). CADD: Predicting the deleteriousness of variants throughout the human genome. Nucleic Acids Res..

[57] Davydov E.V., Goode D.L., Sirota M., Cooper G.M., Sidow A., Batzoglou S. (2010). Identifying a high fraction of the human genome to be under selective constraint using GERP++. PLoS Comput. Biol..

[58] Dong S., Zhao N., Spragins E., Kagda M.S., Li M., Assis P., Jolanki O., Luo Y., Cherry J.M., Boyle A.P. (2023). Annotating and prioritizing human non-coding variants with RegulomeDB v. 2. Nat. Genet..

[59] Consortium G. (2020). The GTEx Consortium atlas of genetic regulatory effects across human tissues. Science.

[60] Noguchi S., Arakawa T., Fukuda S., Furuno M., Hasegawa A., Hori F., Ishikawa-Kato S., Kaida K., Kaiho A., Kanamori-Katayama M. (2017). FANTOM5 CAGE profiles of human and mouse samples. Sci. Data.

[61] Ingolia N.T., Ghaemmaghami S., Newman J.R., Weissman J.S. (2009). Genome-wide analysis in vivo of translation with nucleotide resolution using ribosome profiling. Science.

[62] Livak K.J., Schmittgen T.D. (2001). Analysis of relative gene expression data using real-time quantitative PCR and the 2(-Delta Delta C(T)) Method. Methods.

[63] Clarke L., Zheng-Bradley X., Smith R., Kulesha E., Xiao C., Toneva I., Vaughan B., Preuss D., Leinonen R., Shumway M. (2012). The 1000 Genomes Project: Data management and community access. Nat. Methods.

[64] Auer P.L., Reiner A.P., Wang G., Kang H.M., Abecasis G.R., Altshuler D., Bamshad M.J., Nickerson D.A., Tracy R.P., Rich S.S. (2016). Guidelines for Large-Scale Sequence-Based Complex Trait Association Studies: Lessons Learned from the NHLBI Exome Sequencing Project. Am. J. Hum. Genet..

[65] Chen S., Francioli L.C., Goodrich J.K., Collins R.L., Kanai M., Wang Q., Alföldi J., Watts N.A., Vittal C., Gauthier L.D. (2024). A genomic mutational constraint map using variation in 76,156 human genomes. Nature.

[66] Miller J.B., McKinnon L.M., Whiting M.F., Kauwe J.S.K., Ridge P.G. (2020). Codon Pairs are Phylogenetically Conserved: A comprehensive analysis of codon pairing conservation across the Tree of Life. PLoS ONE.

[67] Miller J.B., McKinnon L.M., Whiting M.F., Ridge P.G. (2020). Codon use and aversion is largely phylogenetically conserved across the tree of life. Mol. Phylogenet. Evol..

[68] Miller J.B., McKinnon L.M., Whiting M.F., Ridge P.G. (2019). CAM: An alignment-free method to recover phylogenies using codon aversion motifs. PeerJ.

[69] Miller J.B., Hippen A.A., Belyeu J.R., Whiting M.F., Ridge P.G. (2017). Missing something? Codon aversion as a new character system in phylogenetics. Cladistics.

[70] Yamaguchi K., Ishigaki K., Suzuki A., Tsuchida Y., Tsuchiya H., Sumitomo S., Nagafuchi Y., Miya F., Tsunoda T., Shoda H. (2022). Splicing QTL analysis focusing on coding sequences reveals mechanisms for disease susceptibility loci. Nat. Commun..

[71] Kudla G., Lipinski L., Caffin F., Helwak A., Zylicz M. (2006). High guanine and cytosine content increases mRNA levels in mammalian cells. PLoS Biol..

[72] Rodriguez A., Wright G., Emrich S., Clark P.L. (2018). %MinMax: A versatile tool for calculating and comparing synonymous codon usage and its impact on protein folding. Protein Sci..

[73] Cannarozzi G., Schraudolph N.N., Faty M., von Rohr P., Friberg M.T., Roth A.C., Gonnet P., Gonnet G., Barral Y. (2010). A role for codon order in translation dynamics. Cell.

[74] Brandman O., Hegde R.S. (2016). Ribosome-associated protein quality control. Nat. Struct. Mol. Biol..

[75] Zhou Z., Sun B., Yu D., Bian M. (2021). Roles of tRNA metabolism in aging and lifespan. Cell Death Dis..

