# Supplementary material for: Ramp Sequence May Explain Synonymous Variant Association with Alzheimer’s Disease in the Paired Immunoglobulin-like Type 2 Receptor Alpha (PILRA)
Source: Biomedicines. 2025 Mar 18;13(3):739. doi: 10.3390/biomedicines13030739 (PMC11940050; doi:10.3390/biomedicines13030739)
Supplement: Supplementary file 1 [file biomedicines-13-00739-s001.zip › biomedicines-3456850-supplementary.pdf]

Supplementary Information for

Ramp Sequence May Explain Synonymous Variant Association with Alzheimer's Disease in the Paired Immunoglobulin-like Type 2 Receptor Alpha (PILRA)

Justin B. Miller [1,2,3,4,*], J. Anthony Brandon [2], Lauren M. Harmon [5], Hady W. Sabra [1,2,3,4], Chloe C. Lucido [1,2,3,4], Josue D. Gonzalez Murcia [5], Kayla A. Nations [2], Samuel H. Payne [5], Mark T. W. Ebbert [2,4,6], John S. K. Kauwe [5] and Perry G. Ridge [5,*]

[1] Department of Pathology and Laboratory Medicine, University of Kentucky, Lexington, KY 40506, USA
[2] Sanders-Brown Center on Aging, University of Kentucky, Lexington, KY 40506, USA
[3] Department of Microbiology, Immunology, and Molecular Genetics, University of Kentucky, Lexington, KY 40506, USA
[4] Division of Biomedical Informatics, Department of Internal Medicine, University of Kentucky, Lexington, KY 40506, USA
[5] Department of Biology, Brigham Young University, Provo, UT 84602, USA
[6] Department of Neuroscience, University of Kentucky, Lexington, KY 40506, USA
* Correspondence: justin.miller@uky.edu (J.B.M.); perry.ridge@byu.edu (P.G.R.)

Table of Contents

Supplementary Tables *3*

Supplementary Table S1: Tissues and Cell Types Without a Ramp Sequence in *PILRA* 3

Supplementary Figures *5*

Supplementary Figure S1: Wildtype Sequence with Annotated Features 5

Supplementary Figure S2: Mutant Sequence with Annotated Features 13

Supplementary Tables

Supplementary Table S1: Tissues and Cell Types Without a Ramp Sequence in *PILRA*

**Tissues without *PILRA* Ramp Sequence**	**Cell Types without *PILRA* Ramp Sequence**
Adipose tissue	Adrenal gland glandular cells
Adrenal gland	Appendix glandular cells
Appendix	B-cells
Basal ganglia	Bone marrow hematopoietic
Bone marrow	Breast glandular
Breast	Breast myoepithelial
Cerebellum	Bronchus respiratory epithelial
Cervix uterine	Cerebellum glandular layer
Endometrium	Cerebellum molecular layer
Epididymis	Cerebral cortex glial
Kidney	Cerebral cortex neuronal
Liver	Cervix uterine squamous epithelial
Lung	Colon endothelial
Lymph node	Colon glandular
Midbrain	Colon peripheral nerve or ganglion
Ovary	Duodenum glandular
Parathyroid gland	Endometrium glandular
Pituitary gland	Endometrium glandular
Placenta	Epididymis glandular
Pons and medulla	Esophagus squamous epithelial
Prostate	Fallopian tube glandular
Rectum	Gallbladder glandular
Smooth muscle	Granulocytes
Spinal cord	Heart muscle myocytes
Substantia nigra	Hippocampus neuronal
Testis	Kidney glomeruli
Thalamus	Kidney tubules
Thymus	Liver hepatocytes
Thyroid gland	Lung macrophages
Tonsil	Lymph node germinal center
Urinary bladder	Nasopharynx respiratory epithelial
Vagina	NK-cells

	Pancreas exocrine glandular
	Parathyroid gland glandular
	Placenta decidual
	Placenta trophoblastic
	Rectum glandular
	Salivary gland glandular
	Skin1 Langerhans
	Skin2 epidermal
	Small intestine glandular
	Stomach1 glandular
	Stomach2 glandular
	T-cells
	Testis cells in seminiferous ducts
	Testis Leydig
	Tonsil germinal center
	Tonsil squamous epithelial
	Urinary bladder urothelial
	Vagina squamous epithelial

Supplementary Figures

Supplementary Figure S1: Wildtype Sequence with Annotated Features

All features and annotations are displayed using SnapGene to illustrate the reference (wildtype) sequence that was transfected into CHO-K1 plasmids. The feature annotations comprise the following seven pages.

The *PILRA* gene sequence starts at position 1034. The *rs2405442:T>C* mutation occurs at position 1067 with the wildtype nucleotide shown in this figure. The rest of the sequence is identical to the mutant.

```
5' AACAAAATATTAACGCTTACAATTTCCATTCGCCATTCAGGCTGCGCAACTGTTGGGAAGGGCGATCGGTGCGGGCCTCTTCGCT   85
3' TTGTTTTATAATTGCGAATGTTAAAGGTAAGCGGTAAGTCCGACGCGTTGACAACCCTTCCCGCTAGCCACGCCCGGAGAAGCGA
   f1 ori

ATTACGCCAGCTGGCGAAAGGGGGATGTGCTGCAAGGCGATTAAGTTGGGTAACGCCAGGGTTTTCCCAGTCACGACGTTGTAAA   170
TAATGCGGTCGACCGCTTTCCCCCTACACGACGTTCCGCTAATTCAACCCATTGCGGTCCCAAAAGGGTCAGTGCTGCAACATTT
M13 fwd

ACGACGGCCAGTGCCAAGCTGATCTATACATTGAATCAATATTGGCAATTAGCCATATTAGTCATTGGTTATATAGCATAAATCA   255
TGCTGCCGGTCACGGTTCGACTAGATATGTAACTTAGTTATAACCGTTAATCGGTATAATCAGTAACCAATATATCGTATTTAGT
M13 fwd

BsrGI
ATATTGGCTATTGGCCATTGCATACGTTGTATCTATATCATAATATGTACATTTATATTGGCTCATGTCCAATATGACCGCCATG   340
TATAACCGATAACCGGTAACGTATGCAACATAGATATAGTATTATACATGTAAATATAACCGAGTACAGGTTATACTGGCGGTAC

SpeI AseI
TTGACATTGATTATTGACTAGTTATTAATAGTAATCAATTACGGGGTCATTAGTTCATAGCCCATATATGGAGTTCCGCGTTACA   425
AACTGTAACTAATAACTGATCAATAATTATCATTAGTTAATGCCCCAGTAATCAAGTATCGGGTATATACCTCAAGGCGCAATGT
CMV enhancer

TAACTTACGGTAAATGGCCCGCCTGGCTGACCGCCCAACGACCCCCGCCCATTGACGTCAATAATGACGTATGTTCCCATAGTAA   510
ATTGAATGCCATTTACCGGGCGGACCGACTGGCGGGTTGCTGGGGGCGGGTAACTGCAGTTATTACTGCATACAAGGGTATCATT
CMV enhancer

CGCCAATAGGGACTTTCCATTGACGTCAATGGGTGGAGTATTTACGGTAAACTGCCCACTTGGCAGTACATCAAGTGTATCATAT   595
GCGGTTATCCCTGAAAGGTAACTGCAGTTACCCACCTCATAAATGCCATTTGACGGGTGAACCGTCATGTAGTTCACATAGTATA
CMV enhancer

GCCAAGTCCGCCCCCTATTGACGTCAATGACGGTAAATGGCCCGCCTGGCATTATGCCCAGTACATGACCTTACGGGACTTTCCT   680
CGGTTCAGGCGGGGGATAACTGCAGTTACTGCCATTTACCGGGCGGACCGTAATACGGGTCATGTACTGGAATGCCCTGAAAGGA
CMV enhancer

SnaBI
ACTTGGCAGTACATCTACGTATTAGTCATCGCTATTACCATGGTGATGCGGTTTTGGCAGTACACCAATGGGCGTGGATAGCGGT   765
TGAACCGTCATGTAGATGCATAATCAGTAGCGATAATGGTACCACTACGCCAAAACCGTCATGTGGTTACCCGCACCTATCGCCA
CMV enhancer   CMV promoter

TTGACTCACGGGGATTTCCAAGTCTCCACCCCATTGACGTCAATGGGAGTTTGTTTTGGCACCAAAATCAACGGGACTTTCCAAA   850
AACTGAGTGCCCCTAAAGGTTCAGAGGTGGGGTAACTGCAGTTACCCTCAAACAAAACCGTGGTTTTAGTTGCCCTGAAAGGTTT
CMV promoter

ATGTCGTAATAACCCCGCCCCGTTGACGCAAATGGGCGGTAGGCGTGTACGGTGGGAGGTCTATATAAGCAGAGCTCGTTTAGTG   935
TACAGCATTATTGGGGCGGGGCAACTGCGTTTACCCGCCATCCGCACATGCCACCCTCCAGATATATTCGTCTCGAGCAAATCAC
CMV promoter
```

```
                                                  EcoRI     SalI AccI    BamHI  Acc65I KpnI  BglII
AACCGTCAGAATTTTGTAATACGACTCACTATAGGGCGGCCGGGAATTCGTCGACTGGATCCGGTACCGAGGAGATCTGCCGCCG   1020
TTGGCAGTCTTAAAACATTATGCTGAGTGATATCCCGCCGGCCCTTAAGCAGCTGACCTAGGCCATGGCTCCTCTAGACGGCGGC
               T7 promoter

 AsiSI
CGATCGCGCCACCatgggtcggcccctgctgctgcccctactgcccttgctgctgccgccagcatttctgcagcctagtggctcc   1105
GCTAGCGCGGTGGtacccagccggggacgacgacggggatgacgggaacgacgacggcggtcgtaaagacgtcggatcaccgagg
PILRA_wt
Kozak sequence                       wt_ttg
Met Gly Arg Pro Leu Leu Leu Pro Leu Leu Pro Leu Leu Leu Pro Pro Ala Phe Leu Gln Pro Ser Gly Ser

acaggatctggtccaagctacctttatggggtcactcaaccaaaacacctctcagcctccatgggtggctctgtggaaatcccct   1190
tgtcctagaccaggttcgatggaaataccccagtgagttggttttgtggagagtcggaggtacccaccgagacacctttagggga
PILRA_wt
Thr Gly Ser Gly Pro Ser Tyr Leu Tyr Gly Val Thr Gln Pro Lys His Leu Ser Ala Ser Met Gly Gly Ser Val Glu Ile Pro

                                         BmgBI
tctccttctattaccccctgggagttagccacagctcccgacgtgagaatatcctggagacggggccacttccacaggcagtcctt   1275
agaggaagataatggggaccctcaatcggtgtcgagggctgcactcttataggacctctgccccggtgaaggtgtccgtcaggaa
PILRA_wt
Phe Ser Phe Tyr Tyr Pro Trp Glu Leu Ala Thr Ala Pro Asp Val Arg Ile Ser Trp Arg Arg Gly His Phe His Arg Gln Ser Phe

ctacagcacaaggccgccttccattcacaaggattatgtgaaccggctctttctgaactggacagagggtcagaagagcggcttc   1360
gatgtcgtgttccggcggaaggtaagtgttcctaatacacttggccgagaaagacttgacctgtctcccagtcttctcgccgaag
PILRA_wt
Tyr Ser Thr Arg Pro Pro Ser Ile His Lys Asp Tyr Val Asn Arg Leu Phe Leu Asn Trp Thr Glu Gly Gln Lys Ser Gly Phe

ctcaggatctccaacctgcagaagcaggaccagtctgtgtatttctgccgagttgagctggacacacggagctcagggaggcagc   1445
gagtcctagaggttggacgtcttcgtcctggtcagacacataaagacggctcaactcgacctgtgtgcctcgagtccctccgtcg
PILRA_wt
Leu Arg Ile Ser Asn Leu Gln Lys Gln Asp Gln Ser Val Tyr Phe Cys Arg Val Glu Leu Asp Thr Arg Ser Ser Gly Arg Gln

agtggcagtccatcgaggggaccaaactctccatcacccaggctgtcacgaccaccacccagaggcccagcagcatgactaccac   1530
tcaccgtcaggtagctcccctggtttgagaggtagtgggtccgacagtgctggtggtgggtctccgggtcgtcgtactgatggtg
PILRA_wt
Gln Trp Gln Ser Ile Glu Gly Thr Lys Leu Ser Ile Thr Gln Ala Val Thr Thr Thr Thr Gln Arg Pro Ser Ser Met Thr Thr Thr

ctggaggctcagtagcacaaccaccacaaccggcctcagggtcacacagggcaaacgacgctcagactcttggcacataagtctg   1615
gacctccgagtcatcgtgttggtggtgttggccggagtcccagtgtgtcccgtttgctgcgagtctgagaaccgtgtattcagac
PILRA_wt
Trp Arg Leu Ser Ser Thr Thr Thr Thr Thr Gly Leu Arg Val Thr Gln Gly Lys Arg Arg Ser Asp Ser Trp His Ile Ser Leu

gagactgctgtgggggtggcagtggctgtcactgtgctcggaatcatgattttgggactgatctgcctcctcaggtggaggagaa   1700
ctctgacgacacccccaccgtcaccgacagtgacacgagccttagtactaaaaccctgactagacggaggagtccacctcctctt
PILRA_wt
Glu Thr Ala Val Gly Val Ala Val Ala Val Thr Val Leu Gly Ile Met Ile Leu Gly Leu Ile Cys Leu Leu Arg Trp Arg Arg
```

```
ggaaaggtcagcagcggactaaagccacaaccccagccagggaacccttccaaaacacagaggagccatatgagaatatcaggaa    1785
cctttccagtcgtcgcctgatttcggtgttggggtcggtcccttgggaaggttttgtgtctcctcggtatactcttatagtcctt
PILRA_wt
Arg Lys Gly Gln Gln Arg Thr Lys Ala Thr Thr Pro Ala Arg Glu Pro Phe Gln Asn Thr Glu Glu Pro Tyr Glu Asn Ile Arg Asn
```

```
tgaaggacaaaatacagatcccaagctaaatcccaaggatgacggcatcgtctatgcttcccttgccctctccagctccacctca    1870
acttcctgttttatgtctagggttcgatttagggttcctactgccgtagcagatacgaagggaacgggagaggtcgaggtggagt
PILRA_wt
Glu Gly Gln Asn Thr Asp Pro Lys Leu Asn Pro Lys Asp Asp Gly Ile Val Tyr Ala Ser Leu Ala Leu Ser Ser Ser Thr Ser
```

```
                                                                    NotI   XhoI / PspXI / PaeR7I
cccagagcacctcccagccaccgtcccctcaagagcccccagaacgagaccctgtactctgtcttaaaggcGCGGCCGCTCGAGC    1955
gggtctcgtggagggtcggtggcaggggagttctcgggggtcttgctctgggacatgagacagaatttccgCGCCGGCGAGCTCG
PILRA_wt                                                                1 Glu Myc
Pro Arg Ala Pro Pro Ser His Arg Pro Leu Lys Ser Pro Gln Asn Glu Thr Leu Tyr Ser Val Leu Lys Ala Arg Pro Leu Glu
```

```
                                       EcoRV                                 PmeI      FseI
AGAAACTCATCTCAGAAGAGGATCTGGCAGCAAATGATATCCTGGATTACAAGGATGACGACGATAAGGTTTAAACGGCCGGCCG    2040
TCTTTGAGTAGAGTCTTCTCCTAGACCGTCGTTTACTATAGGACCTAATGTTCCTACTGCTGCTATTCCAAATTTGCCGGCCGGC
            5                   10                                     1         5
Gln Lys Leu Ile Ser Glu Glu Asp Leu Ala Ala Asn Asp Ile Leu Asp Tyr Lys Asp Asp Asp Asp Lys Val *
Myc                                 (in frame with Myc)              FLAG
Gln Lys Leu Ile Ser Glu Glu Asp Leu Ala Ala Asn Asp Ile Leu Asp Tyr Lys Asp Asp Asp Asp Lys Val *
```

```
SacII                    XmaI / TspMI  SmaI
CGGTCATAGCTGTTTCCTGAACAGATCCCGGGTGGCATCCCTGTGACCCCTCCCCAGTGCCTCTCCTGGCCCTGGAAGTTGCCAC    2125
GCCAGTATCGACAAAGGACTTGTCTAGGGCCCACCGTAGGGACACTGGGGAGGGGTCACGGAGAGGACCGGGACCTTCAACGGTG
M13 rev                           hGH poly(A) signal
```

```
AleI
TCCAGTGCCCACCAGCCTTGTCCTAATAAAATTAAGTTGCATCATTTTGTCTGACTAGGTGTCCTTCTATAATATTATGGGGTGG    2210
AGGTCACGGGTGGTCGGAACAGGATTATTTTAATTCAACGTAGTAAAACAGACTGATCCACAGGAAGATATTATAATACCCCACC
hGH poly(A) signal
```

```
                                         BbsI
AGGGGGGTGGTATGGAGCAAGGGGCAAGTTGGGAAGACAACCTGTAGGGCCTGCGGGGTCTATTGGGAACCAAGCTGGAGTGCAG    2295
TCCCCCCACCATACCTCGTTCCCCGTTCAACCCTTCTGTTGGACATCCCGGACGCCCCAGATAACCCTTGGTTCGACCTCACGTC
hGH poly(A) signal
```

```
              BsgI                                           BbvCI
TGGCACAATCTTGGCTCACTGCAATCTCCGCCTCCTGGGTTCAAGCGATTCTCCTGCCTCAGCCTCCCGAGTTGTTGGGATTCCA    2380
ACCGTGTTAGAACCGAGTGACGTTAGAGGCGGAGGACCCAAGTTCGCTAAGAGGACGGAGTCGGAGGGCTCAACAACCCTAAGGT
hGH poly(A) signal
```

```
               BlpI
GGCATGCATGACCAGGCTCAGCTAATTTTTGTTTTTTTGGTAGAGACGGGGTTTCACCATATTGGCCAGGCTGGTCTCCAACTCC    2465
CCGTACGTACTGGTCCGAGTCGATTAAAAACAAAAAAACCATCTCTGCCCCAAAGTGGTATAACCGGTCCGACCAGAGGTTGAGG
hGH poly(A) signal
```

BstXI

TAATCTCAGGTGATCTACCCACCTTGGCCTCCCAAATTGCTGGGATTACAGGCGTGAACCACTGCTCCCTTCCCTGTCCTTCTGA 2550

ATTAGAGTCCACTAGATGGGTGGAACCGGAGGGTTTAACGACCCTAATGTCCGCACTTGGTGACGAGGGAAGGGACAGGAAGACT

hGH poly(A) signal

AgeI PflMI

TTTTAAAATAACTATACCAGCAGGAGGACGTCCAGACACAGCATAGGCTACCTGGCCATGCCCAACCGGTGGGACATTTGAGTTG 2635

AAAATTTTATTGATATGGTCGTCCTCCTGCAGGTCTGTGTCGTATCCGATGGACCGGTACGGGTTGGCCACCCTGTAAACTCAAC

hGH poly(A) signal

CTTGCTTGGCACTGTCCTCTCATGCGTTGGGTCCACTCAGTAGATGCCTGTTGAATTGGGTACGCGGCCAGCGGCGAGCGGTATC 2720

GAACGAACCGTGACAGGAGAGTACGCAACCCAGGTGAGTCATCTACGGACAACTTAACCCATGCGCCGGTCGCCGCTCGCCATAG

hGH poly(A) signal

PciI
AflIII

AGCTCACTCAAAGGCGGTAATACGGTTATCCACAGAATCAGGGGATAACGCAGGAAAGAACATGTGAGCAAAAGGCCAGCAAAAG 2805

TCGAGTGAGTTTCCGCCATTATGCCAATAGGTGTCTTAGTCCCCTATTGCGTCCTTTCTTGTACACTCGTTTTCCGGTCGTTTTC

GCCAGGAACCGTAAAAAGGCCGCGTTGCTGGCGTTTTTCCATAGGCTCCGCCCCCCTGACGAGCATCACAAAAATCGACGCTCAA 2890

CGGTCCTTGGCATTTTTCCGGCGCAACGACCGCAAAAAGGTATCCGAGGCGGGGGGACTGCTCGTAGTGTTTTTAGCTGCGAGTT

ori

GTCAGAGGTGGCGAAACCCGACAGGACTATAAAGATACCAGGCGTTTCCCCCTGGAAGCTCCCTCGTGCGCTCTCCTGTTCCGAC 2975

CAGTCTCCACCGCTTTGGGCTGTCCTGATATTTCTATGGTCCGCAAAGGGGGACCTTCGAGGGAGCACGCGAGAGGACAAGGCTG

ori

CCTGCCGCTTACCGGATACCTGTCCGCCTTTCTCCCTTCGGGAAGCGTGGCGCTTTCTCATAGCTCACGCTGTAGGTATCTCAGT 3060

GGACGGCGAATGGCCTATGGACAGGCGGAAAGAGGGAAGCCCTTCGCACCGCGAAAGAGTATCGAGTGCGACATCCATAGAGTCA

ori

ApaLI

TCGGTGTAGGTCGTTCGCTCCAAGCTGGGCTGTGTGCACGAACCCCCCGTTCAGCCCGACCGCTGCGCCTTATCCGGTAACTATC 3145

AGCCACATCCAGCAAGCGAGGTTCGACCCGACACACGTGCTTGGGGGGCAAGTCGGGCTGGCGACGCGGAATAGGCCATTGATAG

ori

GTCTTGAGTCCAACCCGGTAAGACACGACTTATCGCCACTGGCAGCAGCCACTGGTAACAGGATTAGCAGAGCGAGGTATGTAGG 3230

CAGAACTCAGGTTGGGCCATTCTGTGCTGAATAGCGGTGACCGTCGTCGGTGACCATTGTCCTAATCGTCTCGCTCCATACATCC

ori

CGGTGCTACAGAGTTCTTGAAGTGGTGGCCTAACTACGGCTACACTAGAAGAACAGTATTTGGTATCTGCGCTCTGCTGAAGCCA 3315

GCCACGATGTCTCAAGAACTTCACCACCGGATTGATGCCGATGTGATCTTCTTGTCATAAACCATAGACGCGAGACGACTTCGGT

ori

GTTACCTTCGGAAAAAGAGTTGGTAGCTCTTGATCCGGCAAACAAACCACCGCTGGTAGCGGTGGTTTTTTTGTTTGCAAGCAGC 3400

CAATGGAAGCCTTTTTCTCAACCATCGAGAACTAGGCCGTTTGTTTGGTGGCGACCATCGCCACCAAAAAAACAAACGTTCGTCG

ori

AGATTACGCGCAGAAAAAAAGGATCTCAAGAAGATCCTTTGATCTTTTCTACGGGGTCTGACGCTCAGTGGAACGAAAACTCACG 3485
TCTAATGCGCGTCTTTTTTTCCTAGAGTTCTTCTAGGAAACTAGAAAAGATGCCCCAGACTGCGAGTCACCTTGCTTTTGAGTGC
ori

TTAAGGGATTTTGGTCATGAGATTATCAAAAAGGATCTTCACCTAGATCCTTTTAAATTAAAAATGAAGTTTTAAATCAATCTAA 3570
AATTCCCTAAAACCAGTACTCTAATAGTTTTTCCTAGAAGTGGATCTAGGAAAATTTAATTTTTACTTCAAAATTTAGTTAGATT

AGTATATATGAGTAACCTGAGGCTATGGCAGGGCCTGCCGCCCCGACGTTGGCTGCGAGCCCTGGGCCTTCACCCGAACTTGGGG 3655
TCATATATACTCATTGGACTCCGATACCGTCCCGGACGGCGGGGCTGCAACCGACGCTCGGGACCCGGAAGTGGGCTTGAACCCC

GGTGGGGTGGGGAAAAGGAAGAAACGCGGGCGTATTGGCCCCAATGGGGTCTCGGTGGGGTATCGACAGAGTGCCAGCCCTGGGA 3740
CCACCCCACCCCTTTTCCTTCTTTGCGCCCGCATAACCGGGGTTACCCCAGAGCCACCCCATAGCTGTCTCACGGTCGGGACCCT

CCGAACCCCGCGTTTATGAACAAACGACCCAACACCGTGCGTTTTATTCTGTCTTTTTATTGCCGTCATAGCGCGGGTTCCTTCC 3825
GGCTTGGGGCGCAAATACTTGTTTGCTGGGTTGTGGCACGCAAAATAAGACAGAAAAATAACGGCAGTATCGCGCCCAAGGAAGG
HSV TK poly(A) signal

GGTATTGTCTCCTTCCGTGTTTCAGTTAGCCTCCCCCTAGGGTGGGCGAAGAACTCCAGCATGAGATCCCCGCGCTGGAGGATCA 3910
CCATAACAGAGGAAGGCACAAAGTCAATCGGAGGGGGATCCCACCCGCTTCTTGAGGTCGTACTCTAGGGGCGCGACCTCCTAGT

TCCAGCCGGCGTCCCGGAAAACGATTCCGAAGCCCAACCTTTCATAGAAGGCGGCGGTGGAATCGAAATCTCGTGATGGCAGGTT 3995
AGGTCGGCCGCAGGGCCTTTTGCTAAGGCTTCGGGTTGGAAAGTATCTTCCGCCGCCACCTTAGCTTTAGAGCACTACCGTCCAA

BstBI

GGGCGTCGCTTGGTCGGTCATTTCGAACCCCAGAGTCCCGCTCAGAAGAACTCGTCAAGAAGGCGATAGAAGGCGATGCGCTGCG 4080
CCCGCAGCGAACCAGCCAGTAAAGCTTGGGGTCTCAGGGCGAGTCTTCTTGAGCAGTTCTTCCGCTATCTTCCGCTACGCGACGC
265 260 255
* Phe Phe Glu Asp Leu Leu Arg Tyr Phe Ala Ile Arg Gln Ser
NeoR/KanR
* Phe Phe Glu Asp Leu Leu Arg Tyr Phe Ala Ile Arg Gln Ser

AATCGGGAGCGGCGATACCGTAAAGCACGAGGAAGCGGTCAGCCCATTCGCCGCCAAGCTCTTCAGCAATATCACGGGTAGCCAA 4165
TTAGCCCTCGCCGCTATGGCATTTCGTGCTCCTTCGCCAGTCGGGTAAGCGGCGGTTCGAGAAGTCGTTATAGTGCCCATCGGTT
250 245 240 235 230 225
Asp Pro Ala Ala Ile Gly Tyr Leu Val Leu Phe Arg Asp Ala Trp Glu Gly Gly Leu Glu Glu Ala Ile Asp Arg Thr Ala Leu
NeoR/KanR
Asp Pro Ala Ala Ile Gly Tyr Leu Val Leu Phe Arg Asp Ala Trp Glu Gly Gly Leu Glu Glu Ala Ile Asp Arg Thr Ala Leu

CGCTATGTCCTGATAGCGATCCGCCACACCCAGCCGGCCACAGTCGATGAATCCAGAAAAGCGGCCATTTTCCACCATGATATTC 4250
GCGATACAGGACTATCGCTAGGCGGTGTGGGTCGGCCGGTGTCAGCTACTTAGGTCTTTTCGCCGGTAAAAGGTGGTACTATAAG
220 215 210 205 200 195
Ala Ile Asp Gln Tyr Arg Asp Ala Val Gly Leu Arg Gly Cys Asp Ile Phe Gly Ser Phe Arg Gly Asn Glu Val Met Ile Asn
NeoR/KanR
Ala Ile Asp Gln Tyr Arg Asp Ala Val Gly Leu Arg Gly Cys Asp Ile Phe Gly Ser Phe Arg Gly Asn Glu Val Met Ile Asn

GGCAAGCAGGCATCGCCATGGGTCACGACGAGATCCTCGCCGTCGGGCATGCTCGCCTTGAGCCTGGCGAACAGTTCGGCTGGCG 4335
CCGTTCGTCCGTAGCGGTACCCAGTGCTGCTCTAGGAGCGGCAGCCCGTACGAGCGGAACTCGGACCGCTTGTCAAGCCGACCGC
190 185 180 175 170
Pro Leu Cys Ala Asp Gly His Thr Val Val Leu Asp Glu Gly Asp Pro Met Ser Ala Lys Leu Arg Ala Phe Leu Glu Ala Pro Ala
NeoR/KanR
Pro Leu Cys Ala Asp Gly His Thr Val Val Leu Asp Glu Gly Asp Pro Met Ser Ala Lys Leu Arg Ala Phe Leu Glu Ala Pro Ala

CGAGCCCCTGATGCTCTTCGTCCAGATCATCCTGATCGACAAGACCGGCTTCCATCCGAGTACGTGCTCGCTCGATGCGATGTTT 4420
GCTCGGGGACTACGAGAAGCAGGTCTAGTAGGACTAGCTGTTCTGGCCGAAGGTAGGCTCATGCACGAGCGAGCTACGCTACAAA
165 160 155 150 145 140
Leu Gly Gln His Glu Glu Asp Leu Asp Asp Gln Asp Val Leu Gly Ala Glu Met Arg Thr Arg Ala Arg Glu Ile Arg His Lys
NeoR/KanR
Leu Gly Gln His Glu Glu Asp Leu Asp Asp Gln Asp Val Leu Gly Ala Glu Met Arg Thr Arg Ala Arg Glu Ile Arg His Lys

CGCTTGGTGGTCGAATGGGCAGGTAGCCGGATCAAGCGTATGCAGCCGCCGCATTGCATCAGCCATGATGGATACTTTCTCGGCA 4505
GCGAACCACCAGCTTACCCGTCCATCGGCCTAGTTCGCATACGTCGGCGGCGTAACGTAGTCGGTACTACCTATGAAAGAGCCGT
135 130 125 120 115 110
Ala Gln His Asp Phe Pro Cys Thr Ala Pro Asp Leu Thr His Leu Arg Arg Met Ala Asp Ala Met Ile Ser Val Lys Glu Ala
NeoR/KanR
Ala Gln His Asp Phe Pro Cys Thr Ala Pro Asp Leu Thr His Leu Arg Arg Met Ala Asp Ala Met Ile Ser Val Lys Glu Ala

PflFI
Tth111I
GGAGCAAGGTGAGATGACAGGAGATCCTGCCCCGGCACTTCGCCCAATAGCAGCCAGTCCCTTCCCGCTTCAGTGACAACGTCGA 4590
CCTCGTTCCACTCTACTGTCCTCTAGGACGGGGCCGTGAAGCGGGTTATCGTCGGTCAGGGAAGGGCGAAGTCACTGTTGCAGCT
105 100 95 90 85
Pro Ala Leu His Ser Ser Leu Leu Asp Gln Gly Pro Val Glu Gly Leu Leu Leu Trp Asp Arg Gly Ala Glu Thr Val Val Asp Leu
NeoR/KanR
Pro Ala Leu His Ser Ser Leu Leu Asp Gln Gly Pro Val Glu Gly Leu Leu Leu Trp Asp Arg Gly Ala Glu Thr Val Val Asp Leu

GCACAGCTGCGCAAGGAACGCCCGTCGTGGCCAGCCACGATAGCCGCGCTGCCTCGTCTTGCAGTTCATTCAGGGCACCGGACAG 4675
CGTGTCGACGCGTTCCTTGCGGGCAGCACCGGTCGGTGCTATCGGCGCGACGGAGCAGAACGTCAAGTAAGTCCCGTGGCCTGTC
80 75 70 65 60 55
Val Ala Ala Cys Pro Val Gly Thr Thr Ala Leu Trp Ser Leu Arg Ala Ala Glu Asp Gln Leu Glu Asn Leu Ala Gly Ser Leu
NeoR/KanR
Val Ala Ala Cys Pro Val Gly Thr Thr Ala Leu Trp Ser Leu Arg Ala Ala Glu Asp Gln Leu Glu Asn Leu Ala Gly Ser Leu

KasI NarI SfoI PluTI
GTCGGTCTTGACAAAAAGAACCGGGCGCCCTGCGCTGACAGCCGGAACACGGCGGCATCAGAGCAGCCGATTGTCTGTTGTGCC 4760
CAGCCAGAACTGTTTTTCTTGGCCCGCGGGGACGCGACTGTCGGCCTTGTGCCGCCGTAGTCTCGTCGGCTAACAGACAACACGG
50 45 40 35 30 25
Asp Thr Lys Val Phe Leu Val Pro Arg Gly Gln Ala Ser Leu Arg Phe Val Ala Ala Asp Ser Cys Gly Ile Thr Gln Gln Ala
NeoR/KanR
Asp Thr Lys Val Phe Leu Val Pro Arg Gly Gln Ala Ser Leu Arg Phe Val Ala Ala Asp Ser Cys Gly Ile Thr Gln Gln Ala

CAGTCATAGCCGAATAGCCTCTCCACCCAAGCGGCCGGAGAACCTGCGTGCAATCCATCTTGTTCAATCATGCGAAACGATCCTC 4845
GTCAGTATCGGCTTATCGGAGAGGTGGGTTCGCCGGCCTCTTGGACGCACGTTAGGTAGAACAAGTTAGTACGCTTTGCTAGGAG
20 15 10 5 1
Trp Asp Tyr Gly Phe Leu Arg Glu Val Trp Ala Ala Pro Ser Gly Ala His Leu Gly Asp Gln Glu Ile Met
NeoR/KanR
Trp Asp Tyr Gly Phe Leu Arg Glu Val Trp Ala Ala Pro Ser Gly Ala His Leu Gly Asp Gln Glu Ile Met

ClaI*
BspDI* StuI SfiI
ATCCTGTCTCTTGATCGATCTTTGCAAAAGCCTAGGCCTCCAAAAAAGCCTCCTCACTACTTCTGGAATAGCTCAGAGGCCGAGG 4930
TAGGACAGAGAACTAGCTAGAAACGTTTTCGGATCCGGAGGTTTTTTCGGAGGAGTGATGAAGACCTTATCGAGTCTCCGGCTCC
SV40 promoter
SV40 ori

```
CGGCCTCGGCCTCTGCATAAATAAAAAAAATTAGTCAGCCATGGGGCGGAGAATGGGCGGAACTGGGCGGAGTTAGGGGCGGGAT
                                                                                      5015
GCCGGAGCCGGAGACGTATTTATTTTTTTTAATCAGTCGGTACCCCGCCTCTTACCCGCCTTGACCCGCCTCAATCCCCGCCCTA
< SV40 promoter
SV40 ori

GGGCGGAGTTAGGGGCGGGACTATGGTTGCTGACTAATTGAGATGCATGCTTTGCATACTTCTGCCTGCTGGGGAGCCTGGGGAC
                                                                                      5100
CCCGCCTCAATCCCCGCCCTGATACCAACGACTGATTAACTCTACGTACGAAACGTATGAAGACGGACGACCCCTCGGACCCCTG
< SV40 promoter

        CsiI
        SexAI*
TTTCCACACCTGGTTGCTGACTAATTGAGATGCATGCTTTGCATACTTCTGCCTGCTGGGGAGCCTGGGGACTTTCCACACCCTA
                                                                                      5185
AAAGGTGTGGACCAACGACTGATTAACTCTACGTACGAAACGTATGAAGACGGACGACCCCTCGGACCCCTGAAAGGTGTGGGAT
< SV40 promoter

ACTGACACACATTCCACAGCTGGTTCTTTCCGCCTCAGGACTCTTCCTTTTTCAATATTATTGAAGCATTTATCAGGGTTATTGT
                                                                                      5270
TGACTGTGTGTAAGGTGTCGACCAAGAAAGGCGGAGTCCTGAGAAGGAAAAAGTTATAATAACTTCGTAAATAGTCCCAATAACA
< SV40 promoter                       < AmpR promoter

CTCATGAGCGGATACATATTTGAATGTATTTAGAAAAATAAACAAATAGGGGTTCCGCGCACATTTCCCCGAAAAGTGCCACCTG
                                                                                      5355
GAGTACTCGCCTATGTATAAACTTACATAAATCTTTTTATTTGTTTATCCCCAAGGCGCGTGTAAAGGGGCTTTTCACGGTGGAC
< AmpR promoter

ACGCGCCCTGTAGCGGCGCATTAAGCGCGGCGGGTGTGGTGGTTACGCGCAGCGTGACCGCTACACTTGCCAGCGCCCTAGCGCC
                                                                                      5440
TGCGCGGGACATCGCCGCGTAATTCGCGCCGCCCACACCACCAATGCGCGTCGCACTGGCGATGTGAACGGTCGCGGGATCGCGG
f1 ori >

CGCTCCTTTCGCTTTCTTCCCTTCCTTTCTCGCCACGTTCGCCGGCTTTCCCCGTCAAGCTCTAAATCGGGGGCTCCCTTTAGGG
                                                                                      5525
GCGAGGAAAGCGAAAGAAGGGAAGGAAAGAGCGGTGCAAGCGGCCGAAAGGGGCAGTTCGAGATTTAGCCCCCGAGGGAAATCCC
f1 ori >

                                                                 DraIII
TTCCGATTTAGTGCTTTACGGCACCTCGACCCCAAAAAACTTGATTAGGGTGATGGTTCACGTAGTGGGCCATCGCCCTGATAGA
                                                                                      5610
AAGGCTAAATCACGAAATGCCGTGGAGCTGGGGTTTTTTGAACTAATCCCACTACCAAGTGCATCACCCGGTAGCGGGACTATCT
f1 ori >

CGGTTTTTCGCCCTTTGACGTTGGAGTCCACGTTCTTTAATAGTGGACTCTTGTTCCAAACTGGAACAACACTCAACCCTATCTC
                                                                                      5695
GCCAAAAAGCGGGAAACTGCAACCTCAGGTGCAAGAAATTATCACCTGAGAACAAGGTTTGACCTTGTTGTGAGTTGGGATAGAG
f1 ori >

                   PsiI
GGTCTATTCTTTTGATTTATAAGGGATTTTGCCGATTTCGGCCTATTGGTTAAAAAATGAGCTGATTTAACAAAAATTTAACGCG
                                                                                      5780
CCAGATAAGAAAACTAAATATTCCCTAAAACGGCTAAAGCCGGATAACCAATTTTTTACTCGACTAAATTGTTTTTAAATTGCGC
f1 ori >

AATTTT   3'
      ...   5786
TTAAAA   5'
f1 ori >
```

Supplementary Figure S2: Mutant Sequence with Annotated Features

All features and annotations are displayed using SnapGene to illustrate the mutant sequence that was transfected into CHO-K1 plasmids. The only difference between the mutant and the wildtype sequence is *rs2405442:T>C*. Feature annotations comprise the following eight pages.

The *PILRA* gene sequence starts at position 1034. The *rs2405442:T>C* mutation occurs at position 1067. The rest of the sequence is identical to the wildtype.

Sequence: PILRA_mt_Cterminaltag.dna (Circular / 5786 bp)
Enzymes: Unique 6+ Cutters (50 of 678 total)
Features: 18 total

Unique Cutters **Bold**

```
5′  AACAAAATATTAACGCTTACAATTTCCATTCGCCATTCAGGCTGCGCAACTGTTGGGAAGGGCGATCGGTGCGGGCCTCT
...                                                                                  80
3′  TTGTTTTATAATTGCGAATGTTAAAGGTAAGCGGTAAGTCCGACGCGTTGACAACCCTTCCCGCTAGCCACGCCCGGAGA
    f1 ori

    TCGCTATTACGCCAGCTGGCGAAAGGGGGATGTGCTGCAAGGCGATTAAGTTGGGTAACGCCAGGGTTTTCCCAGTCACG
                                                                                     160
    AGCGATAATGCGGTCGACCGCTTTCCCCCTACACGACGTTCCGCTAATTCAACCCATTGCGGTCCCAAAAGGGTCAGTGC

    ACGTTGTAAAACGACGGCCAGTGCCAAGCTGATCTATACATTGAATCAATATTGGCAATTAGCCATATTAGTCATTGGTT
                                                                                     240
    TGCAACATTTTGCTGCCGGTCACGGTTCGACTAGATATGTAACTTAGTTATAACCGTTAATCGGTATAATCAGTAACCAA
    M13 fwd

                                                                   BsrGI
    ATATAGCATAAATCAATATTGGCTATTGGCCATTGCATACGTTGTATCTATATCATAATATGTACATTTATATTGGCTCA
                                                                                     320
    TATATCGTATTTAGTTATAACCGATAACCGGTAACGTATGCAACATAGATATAGTATTATACATGTAAATATAACCGAGT

                                        SpeI    AseI
    TGTCCAATATGACCGCCATGTTGACATTGATTATTGACTAGTTATTAATAGTAATCAATTACGGGGTCATTAGTTCATAG
                                                                                     400
    ACAGGTTATACTGGCGGTACAACTGTAACTAATAACTGATCAATAATTATCATTAGTTAATGCCCCAGTAATCAAGTATC
    CMV enhancer

    CCCATATATGGAGTTCCGCGTTACATAACTTACGGTAAATGGCCCGCCTGGCTGACCGCCCAACGACCCCCGCCCATTGA
                                                                                     480
    GGGTATATACCTCAAGGCGCAATGTATTGAATGCCATTTACCGGGCGGACCGACTGGCGGGTTGCTGGGGGCGGGTAACT
    CMV enhancer

    CGTCAATAATGACGTATGTTCCCATAGTAACGCCAATAGGGACTTTCCATTGACGTCAATGGGTGGAGTATTTACGGTAA
                                                                                     560
    GCAGTTATTACTGCATACAAGGGTATCATTGCGGTTATCCCTGAAAGGTAACTGCAGTTACCCACCTCATAAATGCCATT
    CMV enhancer

    ACTGCCCACTTGGCAGTACATCAAGTGTATCATATGCCAAGTCCGCCCCCTATTGACGTCAATGACGGTAAATGGCCCGC
                                                                                     640
    TGACGGGTGAACCGTCATGTAGTTCACATAGTATACGGTTCAGGCGGGGGATAACTGCAGTTACTGCCATTTACCGGGCG
    CMV enhancer

                                                                SnaBI
    CTGGCATTATGCCCAGTACATGACCTTACGGGACTTTCCTACTTGGCAGTACATCTACGTATTAGTCATCGCTATTACCA
                                                                                     720
    GACCGTAATACGGGTCATGTACTGGAATGCCCTGAAAGGATGAACCGTCATGTAGATGCATAATCAGTAGCGATAATGGT
    CMV enhancer

    TGGTGATGCGGTTTTGGCAGTACACCAATGGGCGTGGATAGCGGTTTGACTCACGGGGATTTCCAAGTCTCCACCCCATT
                                                                                     800
    ACCACTACGCCAAAACCGTCATGTGGTTACCCGCACCTATCGCCAAACTGAGTGCCCCTAAAGGTTCAGAGGTGGGGTAA
    CMV promoter
    CMV enhancer

    GACGTCAATGGGAGTTTGTTTTGGCACCAAAATCAACGGGACTTTCCAAAATGTCGTAATAACCCCGCCCCGTTGACGCA
                                                                                     880
    CTGCAGTTACCCTCAAACAAAACCGTGGTTTTAGTTGCCCTGAAAGGTTTTACAGCATTATTGGGGCGGGGCAACTGCGT
    CMV promoter

    AATGGGCGGTAGGCGTGTACGGTGGGAGGTCTATATAAGCAGAGCTCGTTTAGTGAACCGTCAGAATTTTGTAATACGAC
                                                                                     960
    TTACCCGCCATCCGCACATGCCACCCTCCAGATATATTCGTCTCGAGCAAATCACTTGGCAGTCTTAAAACATTATGCTG
    CMV promoter                                                       T7 promoter
```

```
EcoRI  SalI  AccI  BamHI  Acc65I  KpnI  BglII  AsiSI
TCACTATAGGGCGGCCGGGAATTCGTCGACTGGATCCGGTACCGAGGAGATCTGCCGCCGCGATCGCGCCACCatgggtc  1040
AGTGATATCCCGCCGGCCCTTAAGCAGCTGACCTAGGCCATGGCTCCTCTAGACGGCGGCGCTAGCGCGGTGGtacccag
T7 promoter                                                                PILRA_mt
Kozak sequence
Met Gly

ggcccctgctgctgcccctactgcccctgctgctgccgccagcatttctgcagcctagtggctccacaggatctggtcca  1120
ccggggacgacgacggggatgacggggacgacgacggcggtcgtaaagacgtcggatcaccgaggtgtcctagaccaggt
PILRA_mt
mt_ctg
Arg Pro Leu Leu Leu Pro Leu Leu Pro Leu Leu Leu Pro Pro Ala Phe Leu Gln Pro Ser Gly Ser Thr Gly Ser Gly Pro

agctacctttatggggtcactcaaccaaaacacctctcagcctccatgggtggctctgtggaaatccccttctccttcta  1200
tcgatggaaataccccagtgagttggttttgtggagagtcggaggtacccaccgagacacctttaggggaagaggaagat
PILRA_mt
Ser Tyr Leu Tyr Gly Val Thr Gln Pro Lys His Leu Ser Ala Ser Met Gly Gly Ser Val Glu Ile Pro Phe Ser Phe Tyr

BmgBI
ttaccccctgggagttagccacagctcccgacgtgagaatatcctggagacggggccacttccacaggcagtccttctaca  1280
aatggggaccctcaatcggtgtcgagggctgcactcttataggacctctgccccggtgaaggtgtccgtcaggaagatgt
PILRA_mt
Tyr Pro Trp Glu Leu Ala Thr Ala Pro Asp Val Arg Ile Ser Trp Arg Arg Gly His Phe His Arg Gln Ser Phe Tyr

gcacaaggccgccttccattcacaaggattatgtgaaccggctctttctgaactggacagagggtcagaagagcggcttc  1360
cgtgttccggcggaaggtaagtgttcctaatacacttggccgagaaagacttgacctgtctcccagtcttctcgccgaag
PILRA_mt
Ser Thr Arg Pro Pro Ser Ile His Lys Asp Tyr Val Asn Arg Leu Phe Leu Asn Trp Thr Glu Gly Gln Lys Ser Gly Phe

ctcaggatctccaacctgcagaagcaggaccagtctgtgtatttctgccgagttgagctggacacacggagctcagggag  1440
gagtcctagaggttggacgtcttcgtcctggtcagacacataaagacggctcaactcgacctgtgtgcctcgagtccctc
PILRA_mt
Leu Arg Ile Ser Asn Leu Gln Lys Gln Asp Gln Ser Val Tyr Phe Cys Arg Val Glu Leu Asp Thr Arg Ser Ser Gly Arg

gcagcagtggcagtccatcgaggggaccaaactctccatcacccaggctgtcacgaccaccacccagaggcccagcagca  1520
cgtcgtcaccgtcaggtagctcccctggtttgagaggtagtgggtccgacagtgctggtggtgggtctccgggtcgtcgt
PILRA_mt
Gln Gln Trp Gln Ser Ile Glu Gly Thr Lys Leu Ser Ile Thr Gln Ala Val Thr Thr Thr Thr Gln Arg Pro Ser Ser

tgactaccacctggaggctcagtagcacaaccaccacaaccggcctcagggtcacacagggcaaacgacgctcagactct  1600
actgatggtggacctccgagtcatcgtgttggtggtgttggccggagtcccagtgtgtcccgtttgctgcgagtctgaga
PILRA_mt
Met Thr Thr Thr Trp Arg Leu Ser Ser Thr Thr Thr Thr Thr Gly Leu Arg Val Thr Gln Gly Lys Arg Arg Ser Asp Ser
```

tggcacataagtctggagactgctgtgggggtggcagtggctgtcactgtgctcggaatcatgattttgggactgatctg
accgtgtattcagacctctgacgacacccccaccgtcaccgacagtgacacgagccttagtactaaaaccctgactagac 1680
PILRA_mt
Trp His Ile Ser Leu Glu Thr Ala Val Gly Val Ala Val Ala Val Thr Val Leu Gly Ile Met Ile Leu Gly Leu Ile Cys

cctcctcaggtggaggagaaggaaaggtcagcagcggactaaagccacaaccccagccagggaacccttccaaaacacag
ggaggagtccacctcctcttcctttccagtcgtcgcctgatttcggtgttggggtcggtcccttgggaaggttttgtgtc 1760
PILRA_mt
Leu Leu Arg Trp Arg Arg Arg Lys Gly Gln Gln Arg Thr Lys Ala Thr Thr Pro Ala Arg Glu Pro Phe Gln Asn Thr

aggagccatatgagaatatcaggaatgaaggacaaaatacagatcccaagctaaatcccaaggatgacggcatcgtctat
tcctcggtatactcttatagtccttacttcctgttttatgtctagggttcgatttagggttcctactgccgtagcagata 1840
PILRA_mt
Glu Glu Pro Tyr Glu Asn Ile Arg Asn Glu Gly Gln Asn Thr Asp Pro Lys Leu Asn Pro Lys Asp Asp Gly Ile Val Tyr

gcttcccttgccctctccagctccacctcacccagagcacctcccagccaccgtcccctcaagagcccccagaacgagac
cgaagggaacgggagaggtcgaggtggagtgggtctcgtggagggtcggtggcaggggagttctcgggggtcttgctctg 1920
PILRA_mt
Ala Ser Leu Ala Leu Ser Ser Ser Thr Ser Pro Arg Ala Pro Pro Ser His Arg Pro Leu Lys Ser Pro Gln Asn Glu Thr

XhoI
PspXI
NotI PaeR7I EcoRV
cctgtactctgtcttaaaggcGCGGCCGCTCGAGCAGAAACTCATCTCAGAAGAGGATCTGGCAGCAAATGATATCCTGG
ggacatgagacagaatttccgCGCCGGCGAGCTCGTCTTTGAGTAGAGTCTTCTCCTAGACCGTCGTTTACTATAGGACC 2000
1 5 10
Glu Gln Lys Leu Ile Ser Glu Glu Asp Leu Ala Ala Asn Asp Ile Leu
PILRA_mt Myc (in frame with Myc) FLAG
Leu Tyr Ser Val Leu Lys Ala Arg Pro Leu Glu Gln Lys Leu Ile Ser Glu Glu Asp Leu Ala Ala Asn Asp Ile Leu

XmaI
PmeI FseI SacII TspMI SmaI
ATTACAAGGATGACGACGATAAGGTTTAAACGGCCGGCCGCGGTCATAGCTGTTTCCTGAACAGATCCCGGGTGGCATCC
TAATGTTCCTACTGCTGCTATTCCAAATTTGCCGGCCGGCGCCAGTATCGACAAAGGACTTGTCTAGGGCCCACCGTAGG 2080
1 5
Asp Tyr Lys Asp Asp Asp Asp Lys Val *
FLAG M13 rev hGH poly(A) signal
Asp Tyr Lys Asp Asp Asp Asp Lys Val *

AleI
CTGTGACCCCTCCCCAGTGCCTCTCCTGGCCCTGGAAGTTGCCACTCCAGTGCCCACCAGCCTTGTCCTAATAAAATTAA
GACACTGGGGAGGGGTCACGGAGAGGACCGGGACCTTCAACGGTGAGGTCACGGGTGGTCGGAACAGGATTATTTTAATT 2160
hGH poly(A) signal

GTTGCATCATTTTGTCTGACTAGGTGTCCTTCTATAATATTATGGGGTGGAGGGGGGTGGTATGGAGCAAGGGGCAAGTT
CAACGTAGTAAAACAGACTGATCCACAGGAAGATATTATAATACCCCACCTCCCCCCACCATACCTCGTTCCCCGTTCAA 2240
hGH poly(A) signal

BbsI, BsgI
GGGAAGACAACCTGTAGGGCCTGCGGGGTCTATTGGGAACCAAGCTGGAGTGCAGTGGCACAATCTTGGCTCACTGCAAT 2320
CCCTTCTGTTGGACATCCCGGACGCCCCAGATAACCCTTGGTTCGACCTCACGTCACCGTGTTAGAACCGAGTGACGTTA
hGH poly(A) signal

BbvCI, BlpI
CTCCGCCTCCTGGGTTCAAGCGATTCTCCTGCCTCAGCCTCCCGAGTTGTTGGGATTCCAGGCATGCATGACCAGGCTCA 2400
GAGGCGGAGGACCCAAGTTCGCTAAGAGGACGGAGTCGGAGGGCTCAACAACCCTAAGGTCCGTACGTACTGGTCCGAGT
hGH poly(A) signal

GCTAATTTTTGTTTTTTTGGTAGAGACGGGGTTTCACCATATTGGCCAGGCTGGTCTCCAACTCCTAATCTCAGGTGATC 2480
CGATTAAAAACAAAAAAACCATCTCTGCCCCAAAGTGGTATAACCGGTCCGACCAGAGGTTGAGGATTAGAGTCCACTAG
hGH poly(A) signal

BstXI
TACCCACCTTGGCCTCCCAAATTGCTGGGATTACAGGCGTGAACCACTGCTCCCTTCCCTGTCCTTCTGATTTTAAAATA 2560
ATGGGTGGAACCGGAGGGTTTAACGACCCTAATGTCCGCACTTGGTGACGAGGGAAGGGACAGGAAGACTAAAATTTTAT
hGH poly(A) signal

AgeI, PflMI
ACTATACCAGCAGGAGGACGTCCAGACACAGCATAGGCTACCTGGCCATGCCCAACCGGTGGGACATTTGAGTTGCTTGC 2640
TGATATGGTCGTCCTCCTGCAGGTCTGTGTCGTATCCGATGGACCGGTACGGGTTGGCCACCCTGTAAACTCAACGAACG
hGH poly(A) signal

TTGGCACTGTCCTCTCATGCGTTGGGTCCACTCAGTAGATGCCTGTTGAATTGGGTACGCGGCCAGCGGCGAGCGGTATC 2720
AACCGTGACAGGAGAGTACGCAACCCAGGTGAGTCATCTACGGACAACTTAACCCATGCGCCGGTCGCCGCTCGCCATAG
hGH poly(A) signal

PciI
AflIII
AGCTCACTCAAAGGCGGTAATACGGTTATCCACAGAATCAGGGGATAACGCAGGAAAGAACATGTGAGCAAAAGGCCAGC 2800
TCGAGTGAGTTTCCGCCATTATGCCAATAGGTGTCTTAGTCCCCTATTGCGTCCTTTCTTGTACACTCGTTTTCCGGTCG

AAAAGGCCAGGAACCGTAAAAAGGCCGCGTTGCTGGCGTTTTTCCATAGGCTCCGCCCCCCTGACGAGCATCACAAAAAT 2880
TTTTCCGGTCCTTGGCATTTTTCCGGCGCAACGACCGCAAAAAGGTATCCGAGGCGGGGGGACTGCTCGTAGTGTTTTTA
ori

CGACGCTCAAGTCAGAGGTGGCGAAACCCGACAGGACTATAAAGATACCAGGCGTTTCCCCCTGGAAGCTCCCTCGTGCG 2960
GCTGCGAGTTCAGTCTCCACCGCTTTGGGCTGTCCTGATATTTCTATGGTCCGCAAAGGGGGACCTTCGAGGGAGCACGC
ori

CTCTCCTGTTCCGACCCTGCCGCTTACCGGATACCTGTCCGCCTTTCTCCCTTCGGGAAGCGTGGCGCTTTCTCATAGCT 3040
GAGAGGACAAGGCTGGGACGGCGAATGGCCTATGGACAGGCGGAAAGAGGGAAGCCCTTCGCACCGCGAAAGAGTATCGA
ori

ApaLI
CACGCTGTAGGTATCTCAGTTCGGTGTAGGTCGTTCGCTCCAAGCTGGGCTGTGTGCACGAACCCCCCGTTCAGCCCGAC 3120
GTGCGACATCCATAGAGTCAAGCCACATCCAGCAAGCGAGGTTCGACCCGACACACGTGCTTGGGGGGCAAGTCGGGCTG
ori

```
CGCTGCGCCTTATCCGGTAACTATCGTCTTGAGTCCAACCCGGTAAGACACGACTTATCGCCACTGGCAGCAGCCACTGG
                                                                                  3200
GCGACGCGGAATAGGCCATTGATAGCAGAACTCAGGTTGGGCCATTCTGTGCTGAATAGCGGTGACCGTCGTCGGTGACC
<                                    ori

TAACAGGATTAGCAGAGCGAGGTATGTAGGCGGTGCTACAGAGTTCTTGAAGTGGTGGCCTAACTACGGCTACACTAGAA
                                                                                  3280
ATTGTCCTAATCGTCTCGCTCCATACATCCGCCACGATGTCTCAAGAACTTCACCACCGGATTGATGCCGATGTGATCTT
<                                    ori

GAACAGTATTTGGTATCTGCGCTCTGCTGAAGCCAGTTACCTTCGGAAAAAGAGTTGGTAGCTCTTGATCCGGCAAACAA
                                                                                  3360
CTTGTCATAAACCATAGACGCGAGACGACTTCGGTCAATGGAAGCCTTTTTCTCAACCATCGAGAACTAGGCCGTTTGTT
<                                    ori

ACCACCGCTGGTAGCGGTGGTTTTTTTGTTTGCAAGCAGCAGATTACGCGCAGAAAAAAAGGATCTCAAGAAGATCCTTT
                                                                                  3440
TGGTGGCGACCATCGCCACCAAAAAAACAAACGTTCGTCGTCTAATGCGCGTCTTTTTTTCCTAGAGTTCTTCTAGGAAA
<                              ori

GATCTTTTCTACGGGGTCTGACGCTCAGTGGAACGAAAACTCACGTTAAGGGATTTTGGTCATGAGATTATCAAAAAGGA
                                                                                  3520
CTAGAAAAGATGCCCCAGACTGCGAGTCACCTTGCTTTTGAGTGCAATTCCCTAAAACCAGTACTCTAATAGTTTTTCCT

TCTTCACCTAGATCCTTTTAAATTAAAAATGAAGTTTTAAATCAATCTAAAGTATATATGAGTAACCTGAGGCTATGGCA
                                                                                  3600
AGAAGTGGATCTAGGAAAATTTAATTTTTACTTCAAAATTTAGTTAGATTTCATATATACTCATTGGACTCCGATACCGT

GGGCCTGCCGCCCCGACGTTGGCTGCGAGCCCTGGGCCTTCACCCGAACTTGGGGGGTGGGGTGGGGAAAAGGAAGAAAC
                                                                                  3680
CCCGGACGGCGGGGCTGCAACCGACGCTCGGGACCCGGAAGTGGGCTTGAACCCCCCACCCCACCCCTTTTCCTTCTTTG

GCGGGCGTATTGGCCCCAATGGGGTCTCGGTGGGGTATCGACAGAGTGCCAGCCCTGGGACCGAACCCCGCGTTTATGAA
                                                                                  3760
CGCCCGCATAACCGGGGTTACCCCAGAGCCACCCCATAGCTGTCTCACGGTCGGGACCCTGGCTTGGGGCGCAAATACTT
                                                                      HSV TK poly(A) signal

CAAACGACCCAACACCGTGCGTTTTATTCTGTCTTTTTATTGCCGTCATAGCGCGGGTTCCTTCCGGTATTGTCTCCTTC
                                                                                  3840
GTTTGCTGGGTTGTGGCACGCAAAATAAGACAGAAAAATAACGGCAGTATCGCGCCCAAGGAAGGCCATAACAGAGGAAG
            HSV TK poly(A) signal

CGTGTTTCAGTTAGCCTCCCCCTAGGGTGGGCGAAGAACTCCAGCATGAGATCCCCGCGCTGGAGGATCATCCAGCCGGC
                                                                                  3920
GCACAAAGTCAATCGGAGGGGGATCCCACCCGCTTCTTGAGGTCGTACTCTAGGGGCGCGACCTCCTAGTAGGTCGGCCG

GTCCCGGAAAACGATTCCGAAGCCCAACCTTTCATAGAAGGCGGCGGTGGAATCGAAATCTCGTGATGGCAGGTTGGGCG
                                                                                  4000
CAGGGCCTTTTGCTAAGGCTTCGGGTTGGAAAGTATCTTCCGCCGCCACCTTAGCTTTAGAGCACTACCGTCCAACCCGC

                  BstBI
TCGCTTGGTCGGTCATTTCGAACCCCAGAGTCCCGCTCAGAAGAACTCGTCAAGAAGGCGATAGAAGGCGATGCGCTGCG
                                                                                  4080
AGCGAACCAGCCAGTAAAGCTTGGGGTCTCAGGGCGAGTCTTCTTGAGCAGTTCTTCCGCTATCTTCCGCTACGCGACGC
                                   265        260        255
                                   *  Phe Phe Glu Asp Leu Leu Arg Tyr Phe Ala Ile Arg Gln Ser
                                   <              NeoR/KanR
                                   *  Phe Phe Glu Asp Leu Leu Arg Tyr Phe Ala Ile Arg Gln Ser
```

AATCGGGAGCGGCGATACCGTAAAGCACGAGGAAGCGGTCAGCCCATTCGCCGCCAAGCTCTTCAGCAATATCACGGGTA 4160
TTAGCCCTCGCCGCTATGGCATTTCGTGCTCCTTCGCCAGTCGGGTAAGCGGCGGTTCGAGAAGTCGTTATAGTGCCCAT

250 245 240 235 230 225
Asp Pro Ala Ala Ile Gly Tyr Leu Val Leu Phe Arg Asp Ala Trp Glu Gly Gly Leu Glu Glu Ala Ile Asp Arg Thr
NeoR/KanR
Asp Pro Ala Ala Ile Gly Tyr Leu Val Leu Phe Arg Asp Ala Trp Glu Gly Gly Leu Glu Glu Ala Ile Asp Arg Thr

GCCAACGCTATGTCCTGATAGCGATCCGCCACACCCAGCCGGCCACAGTCGATGAATCCAGAAAAGCGGCCATTTTCCAC 4240
CGGTTGCGATACAGGACTATCGCTAGGCGGTGTGGGTCGGCCGGTGTCAGCTACTTAGGTCTTTTCGCCGGTAAAAGGTG

220 215 210 205 200
Ala Leu Ala Ile Asp Gln Tyr Arg Asp Ala Val Gly Leu Arg Gly Cys Asp Ile Phe Gly Ser Phe Arg Gly Asn Glu Val
NeoR/KanR
Ala Leu Ala Ile Asp Gln Tyr Arg Asp Ala Val Gly Leu Arg Gly Cys Asp Ile Phe Gly Ser Phe Arg Gly Asn Glu Val

CATGATATTCGGCAAGCAGGCATCGCCATGGGTCACGACGAGATCCTCGCCGTCGGGCATGCTCGCCTTGAGCCTGGCGA 4320
GTACTATAAGCCGTTCGTCCGTAGCGGTACCCAGTGCTGCTCTAGGAGCGGCAGCCCGTACGAGCGGAACTCGGACCGCT

195 190 185 180 175
Met Ile Asn Pro Leu Cys Ala Asp Gly His Thr Val Val Leu Asp Glu Gly Asp Pro Met Ser Ala Lys Leu Arg Ala Phe
NeoR/KanR
Met Ile Asn Pro Leu Cys Ala Asp Gly His Thr Val Val Leu Asp Glu Gly Asp Pro Met Ser Ala Lys Leu Arg Ala Phe

ACAGTTCGGCTGGCGCGAGCCCCTGATGCTCTTCGTCCAGATCATCCTGATCGACAAGACCGGCTTCCATCCGAGTACGT 4400
TGTCAAGCCGACCGCGCTCGGGGACTACGAGAAGCAGGTCTAGTAGGACTAGCTGTTCTGGCCGAAGGTAGGCTCATGCA

170 165 160 155 150 145
Leu Glu Ala Pro Ala Leu Gly Gln His Glu Glu Asp Leu Asp Asp Gln Asp Val Leu Gly Ala Glu Met Arg Thr Arg
NeoR/KanR
Leu Glu Ala Pro Ala Leu Gly Gln His Glu Glu Asp Leu Asp Asp Gln Asp Val Leu Gly Ala Glu Met Arg Thr Arg

GCTCGCTCGATGCGATGTTTCGCTTGGTGGTCGAATGGGCAGGTAGCCGGATCAAGCGTATGCAGCCGCCGCATTGCATC 4480
CGAGCGAGCTACGCTACAAAGCGAACCACCAGCTTACCCGTCCATCGGCCTAGTTCGCATACGTCGGCGGCGTAACGTAG

140 135 130 125 120
Ala Arg Glu Ile Arg His Lys Ala Gln His Asp Phe Pro Cys Thr Ala Pro Asp Leu Thr His Leu Arg Arg Met Ala Asp
NeoR/KanR
Ala Arg Glu Ile Arg His Lys Ala Gln His Asp Phe Pro Cys Thr Ala Pro Asp Leu Thr His Leu Arg Arg Met Ala Asp

AGCCATGATGGATACTTTCTCGGCAGGAGCAAGGTGAGATGACAGGAGATCCTGCCCCGGCACTTCGCCCAATAGCAGCC 4560
TCGGTACTACCTATGAAAGAGCCGTCCTCGTTCCACTCTACTGTCCTCTAGGACGGGGCCGTGAAGCGGGTTATCGTCGG

115 110 105 100 95
Ala Met Ile Ser Val Lys Glu Ala Pro Ala Leu His Ser Ser Leu Leu Asp Gln Gly Pro Val Glu Gly Leu Leu Leu Trp
NeoR/KanR
Ala Met Ile Ser Val Lys Glu Ala Pro Ala Leu His Ser Ser Leu Leu Asp Gln Gly Pro Val Glu Gly Leu Leu Leu Trp

PflFI
Tth111I

AGTCCCTTCCCGCTTCAGTGACAACGTCGAGCACAGCTGCGCAAGGAACGCCCGTCGTGGCCAGCCACGATAGCCGCGCT 4640
TCAGGGAAGGGCGAAGTCACTGTTGCAGCTCGTGTCGACGCGTTCCTTGCGGGCAGCACCGGTCGGTGCTATCGGCGCGA

90 85 80 75 70 65
Asp Arg Gly Ala Glu Thr Val Val Asp Leu Val Ala Ala Cys Pro Val Gly Thr Thr Ala Leu Trp Ser Leu Arg Ala
NeoR/KanR
Asp Arg Gly Ala Glu Thr Val Val Asp Leu Val Ala Ala Cys Pro Val Gly Thr Thr Ala Leu Trp Ser Leu Arg Ala

KasI NarI SfoI PluTI

```
GCCTCGTCTTGCAGTTCATTCAGGGCACCGGACAGGTCGGTCTTGACAAAAAGAACCGGGCGCCCCTGCGCTGACAGCCG   4720
CGGAGCAGAACGTCAAGTAAGTCCCGTGGCCTGTCCAGCCAGAACTGTTTTTCTTGGCCCGCGGGGACGCGACTGTCGGC
```

60 55 50 45 40
Ala Glu Asp Gln Leu Glu Asn Leu Ala Gly Ser Leu Asp Thr Lys Val Phe Leu Val Pro Arg Gly Gln Ala Ser Leu Arg
NeoR/KanR
Ala Glu Asp Gln Leu Glu Asn Leu Ala Gly Ser Leu Asp Thr Lys Val Phe Leu Val Pro Arg Gly Gln Ala Ser Leu Arg

```
GAACACGGCGGCATCAGAGCAGCCGATTGTCTGTTGTGCCCAGTCATAGCCGAATAGCCTCTCCACCCAAGCGGCCGGAG   4800
CTTGTGCCGCCGTAGTCTCGTCGGCTAACAGACAACACGGGTCAGTATCGGCTTATCGGAGAGGTGGGTTCGCCGGCCTC
```

35 30 25 20 15
Phe Val Ala Ala Asp Ser Cys Gly Ile Thr Gln Gln Ala Trp Asp Tyr Gly Phe Leu Arg Glu Val Trp Ala Ala Pro Ser
NeoR/KanR
Phe Val Ala Ala Asp Ser Cys Gly Ile Thr Gln Gln Ala Trp Asp Tyr Gly Phe Leu Arg Glu Val Trp Ala Ala Pro Ser

ClaI* BspDI*

```
AACCTGCGTGCAATCCATCTTGTTCAATCATGCGAAACGATCCTCATCCTGTCTCTTGATCGATCTTTGCAAAAGCCTAG   4880
TTGGACGCACGTTAGGTAGAACAAGTTAGTACGCTTTGCTAGGAGTAGGACAGAGAACTAGCTAGAAACGTTTTCGGATC
```

10 5 1
Gly Ala His Leu Gly Asp Gln Glu Ile Met
NeoR/KanR
SV40 promoter
SV40 ori
Gly Ala His Leu Gly Asp Gln Glu Ile Met

StuI SfiI

```
GCCTCCAAAAAAGCCTCCTCACTACTTCTGGAATAGCTCAGAGGCCGAGGCGGCCTCGGCCTCTGCATAAATAAAAAAAA   4960
CGGAGGTTTTTTCGGAGGAGTGATGAAGACCTTATCGAGTCTCCGGCTCCGCCGGAGCCGGAGACGTATTTATTTTTTTT
```

SV40 promoter
SV40 ori

```
TTAGTCAGCCATGGGGCGGAGAATGGGCGGAACTGGGCGGAGTTAGGGGCGGGATGGGCGGAGTTAGGGGCGGGACTATG   5040
AATCAGTCGGTACCCCGCCTCTTACCCGCCTTGACCCGCCTCAATCCCCGCCCTACCCGCCTCAATCCCCGCCCTGATAC
```

SV40 promoter
SV40 ori

CsiI SexAI*

```
GTTGCTGACTAATTGAGATGCATGCTTTGCATACTTCTGCCTGCTGGGGAGCCTGGGGACTTTCCACACCTGGTTGCTGA   5120
CAACGACTGATTAACTCTACGTACGAAACGTATGAAGACGGACGACCCCTCGGACCCCTGAAAGGTGTGGACCAACGACT
```

SV40 promoter

```
CTAATTGAGATGCATGCTTTGCATACTTCTGCCTGCTGGGGAGCCTGGGGACTTTCCACACCCTAACTGACACACATTCC   5200
GATTAACTCTACGTACGAAACGTATGAAGACGGACGACCCCTCGGACCCCTGAAAGGTGTGGGATTGACTGTGTGTAAGG
```

SV40 promoter

```
ACAGCTGGTTCTTTCCGCCTCAGGACTCTTCCTTTTTCAATATTATTGAAGCATTTATCAGGGTTATTGTCTCATGAGCG   5280
TGTCGACCAAGAAAGGCGGAGTCCTGAGAAGGAAAAAGTTATAATAACTTCGTAAATAGTCCCAATAACAGAGTACTCGC
```

SV40 promoter AmpR promoter

```
GATACATATTTGAATGTATTTAGAAAAATAAACAAATAGGGGTTCCGCGCACATTTCCCCGAAAAGTGCCACCTGACGCG
                                                                                     5360
CTATGTATAAACTTACATAAATCTTTTTATTTGTTTATCCCCAAGGCGCGTGTAAAGGGGCTTTTCACGGTGGACTGCGC
< AmpR promoter                                                           f1 ori >

CCCTGTAGCGGCGCATTAAGCGCGGCGGGTGTGGTGGTTACGCGCAGCGTGACCGCTACACTTGCCAGCGCCCTAGCGCC
                                                                                     5440
GGGACATCGCCGCGTAATTCGCGCCGCCCACACCACCAATGCGCGTCGCACTGGCGATGTGAACGGTCGCGGGATCGCGG
f1 ori >

CGCTCCTTTCGCTTTCTTCCCTTCCTTTCTCGCCACGTTCGCCGGCTTTCCCCGTCAAGCTCTAAATCGGGGGCTCCCTT
                                                                                     5520
GCGAGGAAAGCGAAAGAAGGGAAGGAAAGAGCGGTGCAAGCGGCCGAAAGGGGCAGTTCGAGATTTAGCCCCCGAGGGAA
f1 ori >

                                                                      DraIII
TAGGGTTCCGATTTAGTGCTTTACGGCACCTCGACCCCAAAAAACTTGATTAGGGTGATGGTTCACGTAGTGGGCCATCG
                                                                                     5600
ATCCCAAGGCTAAATCACGAAATGCCGTGGAGCTGGGGTTTTTTGAACTAATCCCACTACCAAGTGCATCACCCGGTAGC
f1 ori >

CCCTGATAGACGGTTTTTCGCCCTTTGACGTTGGAGTCCACGTTCTTTAATAGTGGACTCTTGTTCCAAACTGGAACAAC
                                                                                     5680
GGGACTATCTGCCAAAAAGCGGGAAACTGCAACCTCAGGTGCAAGAAATTATCACCTGAGAACAAGGTTTGACCTTGTTG
f1 ori >

                                    PsiI
ACTCAACCCTATCTCGGTCTATTCTTTTGATTTATAAGGGATTTTGCCGATTTCGGCCTATTGGTTAAAAAATGAGCTGA
                                                                                     5760
TGAGTTGGGATAGAGCCAGATAAGAAAACTAAATATTCCCTAAAACGGCTAAAGCCGGATAACCAATTTTTTACTCGACT
f1 ori >

TTTAACAAAAATTTAACGCGAATTTT   3'
                         ...  5786
AAATTGTTTTTAAATTGCGCTTAAAA   5'
f1 ori >
```
